# Supplementary material for: Loss of p53 activates thyroid hormone via type 2 deiodinase and enhances DNA damage
Source: Nat Commun. 2023 Mar 4;14:1244. doi: 10.1038/s41467-023-36755-y (PMC9985592; doi:10.1038/s41467-023-36755-y)

Supplementary Information

Loss of p53 activates Thyroid Hormone via type 2 deiodinase and enhances DNA damage

Annarita Nappi[1], Caterina Miro[1], Antonio Pezone[2], Alfonso Tramontano[3], Emery Di Cicco[1], Serena Sagliocchi[1], Annunziata Gaetana Cicatiello[1], Melania Murolo[1], Sepehr Torabinejad[1], Elena Abbotto[4], Giuseppina Caiazzo[1], Maddalena Raia[5], Mariano Stornaiuolo[6], Dario Antonini[2], Gabriella Fabbrocini[1], Domenico Salvatore[5,7], Vittorio Enrico Avvedimento[8] and Monica Dentice[1,5]

[1]Department of Clinical Medicine and Surgery, University of Naples "Federico II", 80131, Naples, Italy; [2]Department of Biology, University of Naples "Federico II", 80126, Naples, Italy; [3]Department of Precision Medicine, University of Campania "L. Vanvitelli", 80138, Naples, Italy; [4]Department of Experimental Medicine, University of Genoa, 16132, Genoa, Italy; [5]CEINGE, Biotecnologie Avanzate S.c.a.r.l., 80131, Naples, Italy; [6]Department of Pharmacy, University of Naples "Federico II", 80149, Naples, Italy; [7]Department of Public Health, University of Naples "Federico II", 80131, Naples, Italy; [8]Department of Molecular Medicine and Medical Biotechnology, University of Naples "Federico II", 80131, Naples, Italy.

a

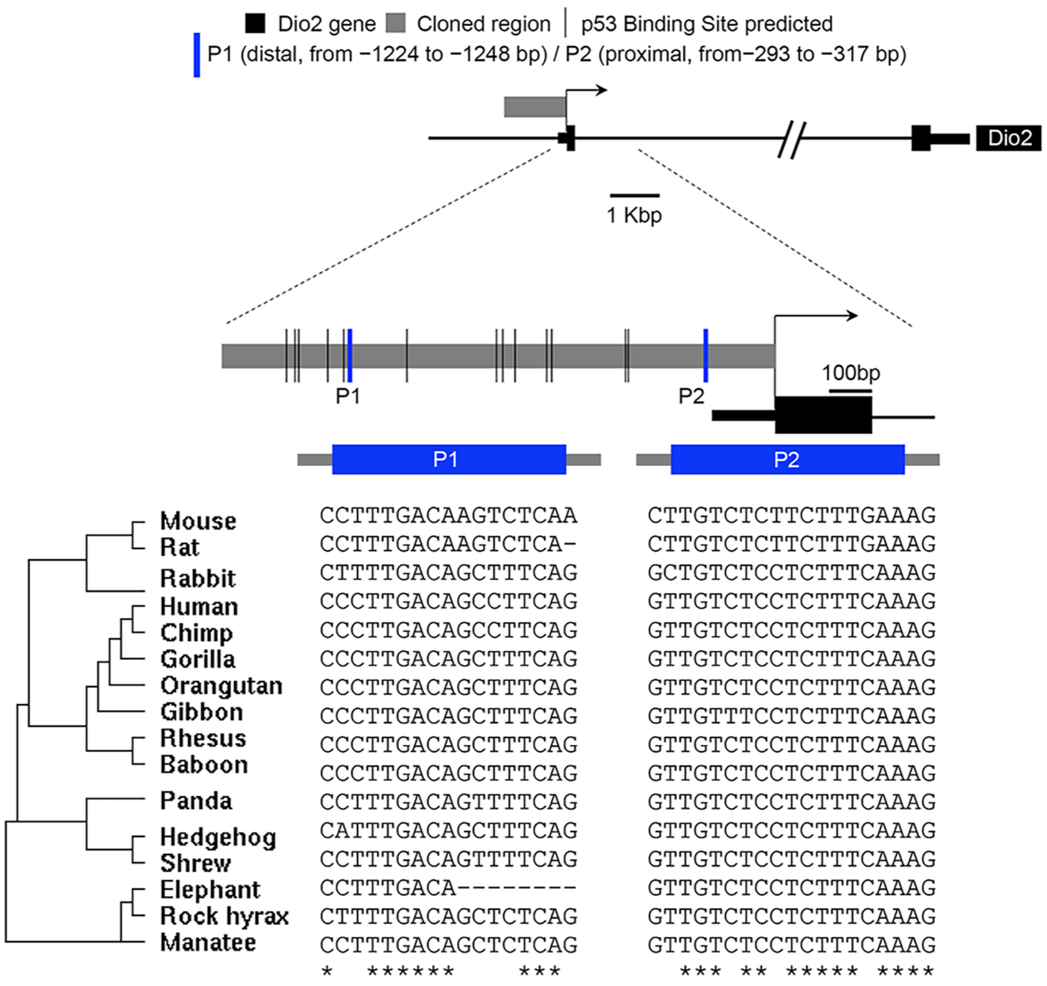

b

AC	ID	Score	Loc.	Str.	Consensus Sequence	Signal Sequence
M00272	V$P53_02	0.805686	127	(+)	NGRCWTGYCY	ACCCATGTTC
M00272	V$P53_02	0.843200	151	(+)	NGRCWTGYCY	CTACCTGCCT
M00272	V$P53_02	0.795135	155	(+)	NGRCWTGYCY	CTGCCTGCCA
M00272	V$P53_02	0.805100	222	(-)	NGRCWTGYCY	TAACATATTA
M00272	V$P53_02	0.819754	261	(+)	NGRCWTGYCY	AAGCATTTCT
M00272	V$P53_02	0.842614	261	(-)	NGRCWTGYCY	AAGCATTTCT
**M00272**	**V$P53_02**	**0.944900**	**273**	**(+)**	**NGRCWTGYCY**	**AGACTTGTCA**
**M00272**	**V$P53_02**	**0.936108**	**273**	**(-)**	**NGRCWTGYCY**	**AGACTTGTCA**
M00272	V$P53_02	0.817116	406	(+)	NGRCWTGYCY	AAGTAAGCCC
M00272	V$P53_02	0.800117	406	(-)	NGRCWTGYCY	AAGTAAGCCC
M00272	V$P53_02	0.825029	624	(-)	NGRCWTGYCY	GGACAGGCAG
M00272	V$P53_02	0.814185	638	(+)	NGRCWTGYCY	AGACAGGCTG
M00272	V$P53_02	0.871043	638	(-)	NGRCWTGYCY	AGACAGGCTG
M00272	V$P53_02	0.850821	664	(+)	NGRCWTGYCY	AAGTATGTTT
M00272	V$P53_02	0.805393	664	(-)	NGRCWTGYCY	AAGTATGTTT
M00272	V$P53_02	0.854631	739	(-)	NGRCWTGYCY	AAACAAACCA
M00272	V$P53_02	0.801583	748	(-)	NGRCWTGYCY	AAACAACCCC
M00272	V$P53_02	0.792790	929	(-)	NGRCWTGYCY	GGACTGGCAC
M00272	V$P53_02	0.806565	933	(+)	NGRCWTGYCY	TGGCACGCCA
M00272	V$P53_02	0.883646	933	(-)	NGRCWTGYCY	TGGCACGCCA
**M00272**	**V$P53_02**	**0.854924**	**1113**	**(+)**	**NGRCWTGYCY**	**AGACAAGTTG**
**M00272**	**V$P53_02**	**0.916471**	**1113**	**(-)**	**NGRCWTGYCY**	**AGACAAGTTG**

Supplementary Fig. 1 | p53 negatively regulates Type 2 deiodinase expression. a, Schematic localization of p53 binding site within the *Dio2* promoter region and conservation analysis of the p53 binding motif. **b**, Consensus sequence analysis of the 15 different putative consensus "half-site" motifs for p53 (ID V$P53_02, AC M00272) within the *Dio2* promoter region.

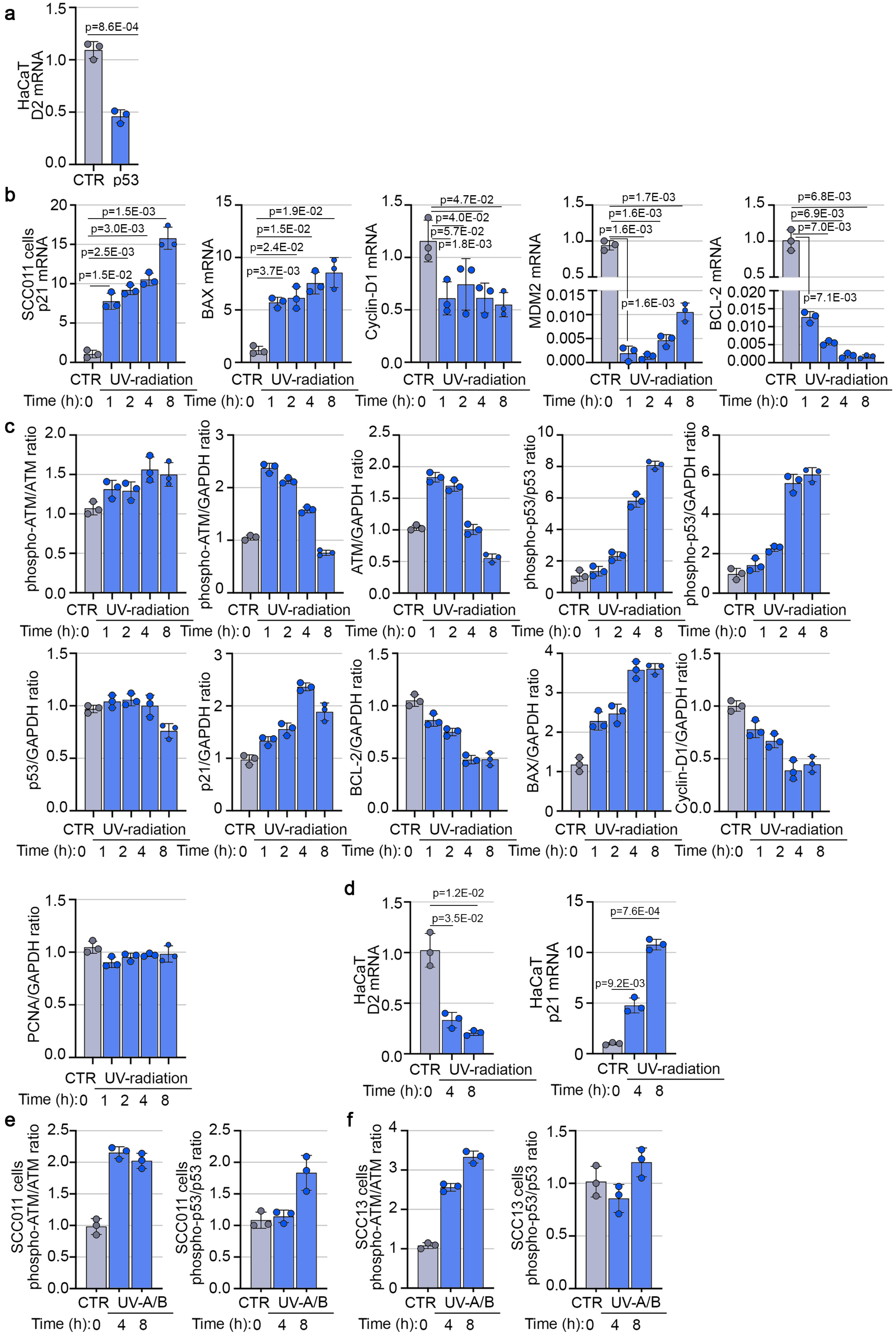

Supplementary Fig. 2 | p53 activation induced by UV-radiation reduces D2 expression. a, D2 mRNA expression was measured by Real-Time PCR in HaCaT cells transiently transfected with a

p53-expressing vector or a control empty plasmid (CTR) (n = 3 independent experiments). **b**, mRNA expression of p53 target genes, namely p21, BAX, Cyclin-D1, MDM-2 and BCL-2, was measured by Real-Time PCR in SCC011 cells, in a time course response to DNA damage induced by cellular exposure to UV-radiation (100 μJ/cm^2) at the indicated time points (0, 1, 2, 4 and 8 hours) (n = 3 independent experiments). All the results are shown as means ± SD from at least 3 separate experiments. p-values were determined by two-tailed Student's t-test. **c**, Quantification of the single protein levels *versus* GAPDH of Western Blot analysis in Fig. 2h are represented by histograms (n = 3 independent experiments). Data are shown as mean ± SD of 3 independent experiments. **d**, D2 and p21 mRNA expression was measured by Real-Time PCR in HaCaT cells in a time course response to DNA damage induced by cellular exposure to UV-radiation (100 μJ/cm^2) at the indicated time points (0, 4 and 8 hours) (n = 3 independent experiments). **e**, **f** Quantification of the single protein levels of Western Blot analysis in Fig. 2k (**e**) and Fig. 2l (**f**) are represented by histograms (n = 3 independent experiments). Data are shown as mean ± SD of 3 independent experiments. Source data are provided as a Source Data file.

a hDIO1_promoter

AC	ID	Score	Loc.	Str.	Consensus Sequence	Signal Sequence
M00272	V$P53_02	0.825615	16	(+)	NGRCWTGYCY	AGCCATGCTG
M00272	V$P53_02	0.808030	16	(-)	NGRCWTGYCY	AGCCATGCTG
M00272	V$P53_02	0.796893	96	(+)	NGRCWTGYCY	CTGCTTGCCG
M00272	V$P53_02	0.832649	152	(+)	NGRCWTGYCY	TGACTTCTCT
M00272	V$P53_02	0.895369	252	(+)	NGRCWTGYCY	TTACTTGTCT
M00272	V$P53_02	0.809203	280	(+)	NGRCWTGYCY	TGACTTCCTT
M00272	V$P53_02	0.804513	340	(+)	NGRCWTGYCY	TTGCAGGCCC
M00272	V$P53_02	0.790152	488	(-)	NGRCWTGYCY	GAGCCTGTAA
M00272	V$P53_02	0.899472	551	(+)	NGRCWTGYCY	AGACCAGCCT
M00272	V$P53_02	0.885991	551	(-)	NGRCWTGYCY	AGACCAGCCT
M00272	V$P53_02	0.834408	575	(+)	NGRCWTGYCY	AACCCTGTCT
M00272	V$P53_02	0.799238	617	(-)	NGRCWTGYCY	TGGCAGGCGC
M00272	V$P53_02	0.805393	812	(+)	NGRCWTGYCY	AAACATCTTT
M00272	V$P53_02	0.806858	812	(-)	NGRCWTGYCY	AAACATCTTT
M00272	V$P53_02	0.804220	874	(+)	NGRCWTGYCY	TGACTTGTGA
M00272	V$P53_02	0.812720	874	(-)	NGRCWTGYCY	TGACTTGTGA
M00272	V$P53_02	0.827081	893	(+)	NGRCWTGYCY	TAACAGGTTC
M00272	V$P53_02	0.833529	893	(-)	NGRCWTGYCY	TAACAGGTTC
M00272	V$P53_02	0.871923	925	(+)	NGRCWTGYCY	GTACTTGTTT
M00272	V$P53_02	0.834115	1033	(-)	NGRCWTGYCY	AAACAGACCT
M00272	V$P53_02	0.821219	1082	(-)	NGRCWTGYCY	AGGCAAACAT
M00272	V$P53_02	0.796307	1086	(+)	NGRCWTGYCY	AAACATCTTC
M00272	V$P53_02	0.791032	1086	(-)	NGRCWTGYCY	AAACATCTTC
M00272	V$P53_02	0.816237	1096	(+)	NGRCWTGYCY	TGACCTGACT

b hDIO3_promoter

AC	ID	Score	Loc.	Str.	Consensus Sequence	Signal Sequence
M00272	V$P53_02	0.794842	20	(+)	NGRCWTGYCY	GGGCCGGCCA
M00272	V$P53_02	0.824736	20	(-)	NGRCWTGYCY	GGGCCGGCCA
M00272	V$P53_02	0.825029	46	(+)	NGRCWTGYCY	GGAGTTGCCC
M00272	V$P53_02	0.793083	103	(+)	NGRCWTGYCY	TGACGCGTCC
M00272	V$P53_02	0.858734	263	(-)	NGRCWTGYCY	AGGCAAGCGG
M00272	V$P53_02	0.801290	701	(+)	NGRCWTGYCY	GGGCGGGCTC
M00272	V$P53_02	0.835580	732	(+)	NGRCWTGYCY	GGGCGAGCCA
M00272	V$P53_02	0.820340	732	(-)	NGRCWTGYCY	GGGCGAGCCA
M00272	V$P53_02	0.814185	760	(+)	NGRCWTGYCY	AAAGTTGCCT
M00272	V$P53_02	0.790445	1120	(-)	NGRCWTGYCY	GAGCTGGCTG
M00272	V$P53_02	0.824736	1138	(-)	NGRCWTGYCY	GGGCCCGCCC

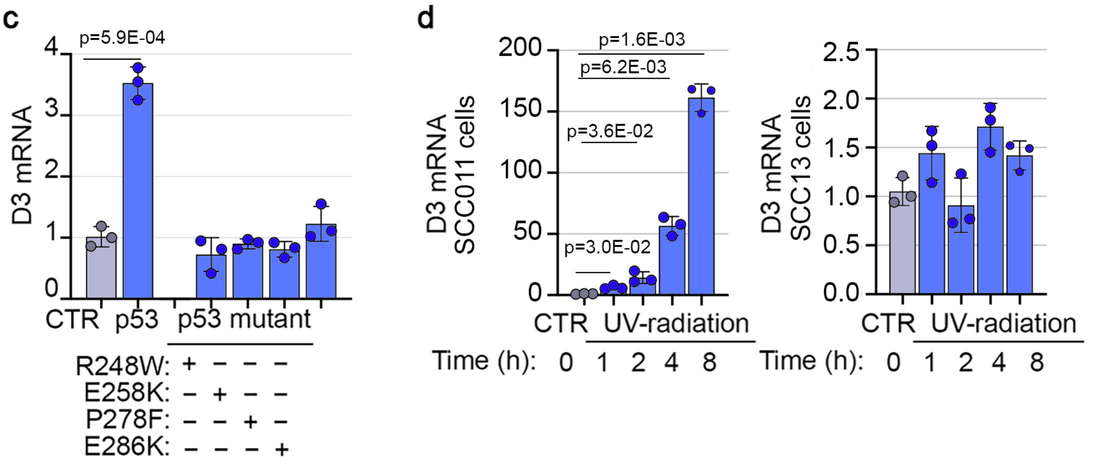

Supplementary Fig. 3 | p53 positively regulates Type 3 deiodinase expression. a, **b**, Analysis of the putative consensus "half-site" motifs for p53 (ID V$P53_02, AC M00272) within the *DIO1* (**a**) and *DIO3* (**b**) promoter region. **c**, D3 mRNA expression was measured by Real-Time PCR in SCC011 cells transiently transfected with four DNA-contact mutant p53 plasmids, carrying different p53 DNA-mutations (R248W, E258K, P278F and E286K). **d**, D3 mRNA expression was measured by Real-Time PCR in SCC011 and SCC13 cells, in a time course response to DNA damage induced by exposure to UV-C radiation (100 µJ/cm^2) at the indicated time points (0, 1, 2, 4 and 8 hours). All the results in Fig. 3 c,d are shown as means ± SD from at least 3 separate experiments. p-values were determined by two-tailed Student's t-test. Source data are provided as a Source Data file.

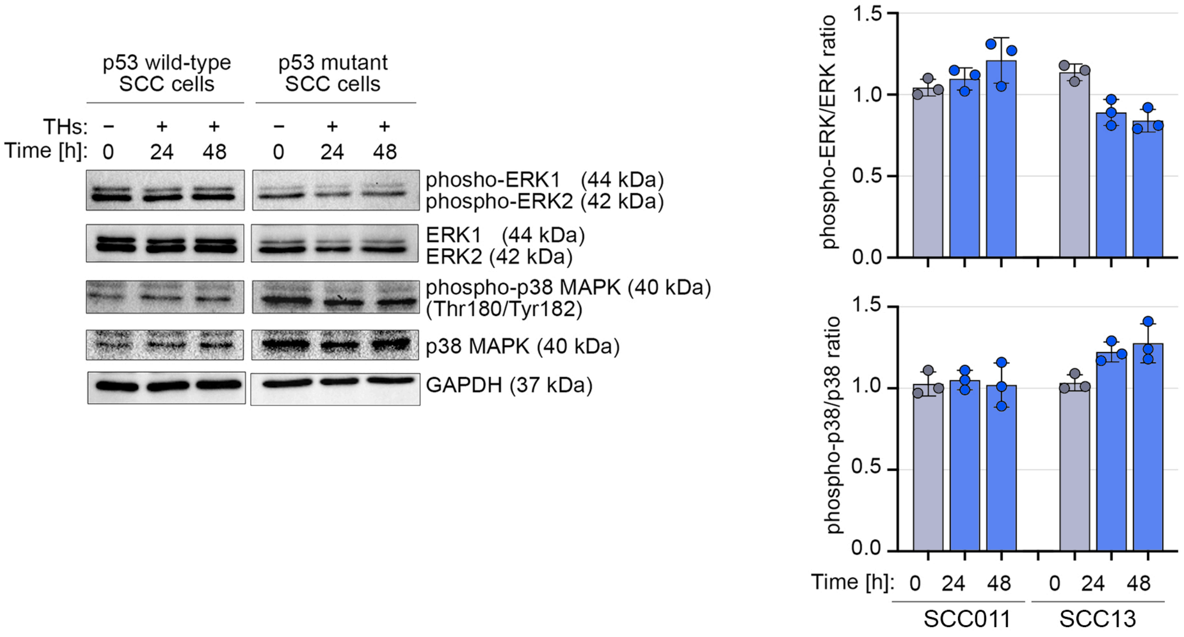

Supplementary Fig. 4 | THs act mainly by their genomic action in SCC cells. Western Blot analysis of phospho-ERK 1/2 and phospho-p38 MAPK in SCC011 and SCC13 cells treated or not with THs at 0, 24 and 48 hours. ERK 1/2, p38 MAPK and GAPDH expression was measured as a loading control. Quantification of the protein levels of phospho-ERK 1/2 versus ERK 1/2 and phospho-p38 MAPK versus p38 MAPK levels are represented by histograms. Data are shown as means ± SD from represent the mean of 3 independent experiments (n = 3 independent experiments). p-values were determined by two-tailed Student's t-test. Source data are provided as a Source Data file.

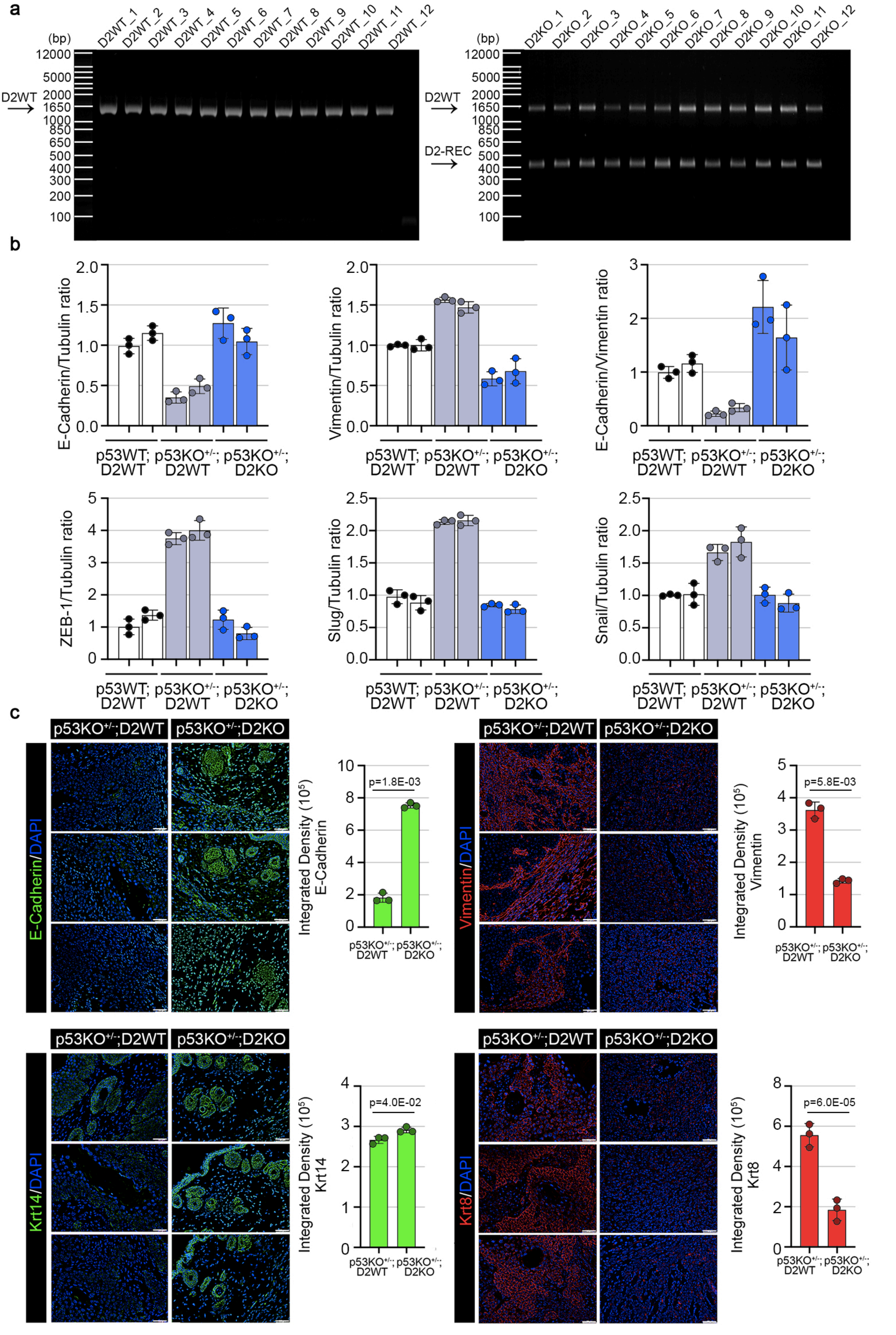
a
(bp)
D2WT_1 D2WT_2 D2WT_3 D2WT_4 D2WT_5 D2WT_6 D2WT_7 D2WT_8 D2WT_9 D2WT_10 D2WT_11 D2WT_12
12000 5000 2000 1650 1000 850 650 500 400 300 200 100
D2WT
D2KO_1 D2KO_2 D2KO_3 D2KO_4 D2KO_5 D2KO_6 D2KO_7 D2KO_8 D2KO_9 D2KO_10 D2KO_11 D2KO_12
D2-REC
b
E-Cadherin/Tubulin ratio
Vimentin/Tubulin ratio
E-Cadherin/Vimentin ratio
ZEB-1/Tubulin ratio
Slug/Tubulin ratio
Snail/Tubulin ratio
p53WT; D2WT
p53KO+/-; D2WT
p53KO+/-; D2KO
c
p53KO+/-;D2WT
p53KO+/-;D2KO
E-Cadherin/DAPI
Vimentin/DAPI
Krt14/DAPI
Krt8/DAPI
Integrated Density (10^5) E-Cadherin
Integrated Density (10^5) Vimentin
Integrated Density (10^5) Krt14
Integrated Density (10^5) Krt8
p=1.8E-03
p=5.8E-03
p=4.0E-02
p=6.0E-05

Supplementary Fig. 5 | *Dio2* depletion in the epidermal compartment attenuates the p53-driven tumorigenesis. a, *Dio2* genetic depletion in epidermal compartment was confirmed by PCR analysis of the dorsal skin sections of p53KO$^{+/-}$;D2KO and p53KO$^{+/-}$;D2WT mice, these latter used as *Dio2* control mice. D2WT band indicate the full length D2 and D2-REC indicates the recombinant D2 fragment generated by the CRE Recombinase (n = 12 mice). **b**, Quantification of the single protein levels *versus* Tubulin of Western Blot analysis in Fig. 3k are represented by histograms (n = 3 independent experiments). Data are shown as mean ± SD of 3 independent experiments. **c**, IF staining with epithelial (E-Cadherin, Krt14) and mesenchymal (Vimentin, Krt8) markers of skin lesions from i) p53KO$^{+/-}$;D2WT and ii) p53KO$^{+/-}$;D2KO mice . Magnification 20X. Scale bars represent 50 µm. Relative quantification of the single immunofluorescent signals (Integrated Density) are represented by histograms (representative of 3 images per sample, n = 3 mice). All the results are shown as means ± SD from at least 3 separate experiments. p-values were determined by two-tailed Student's t-test. Source data are provided as a Source Data file.

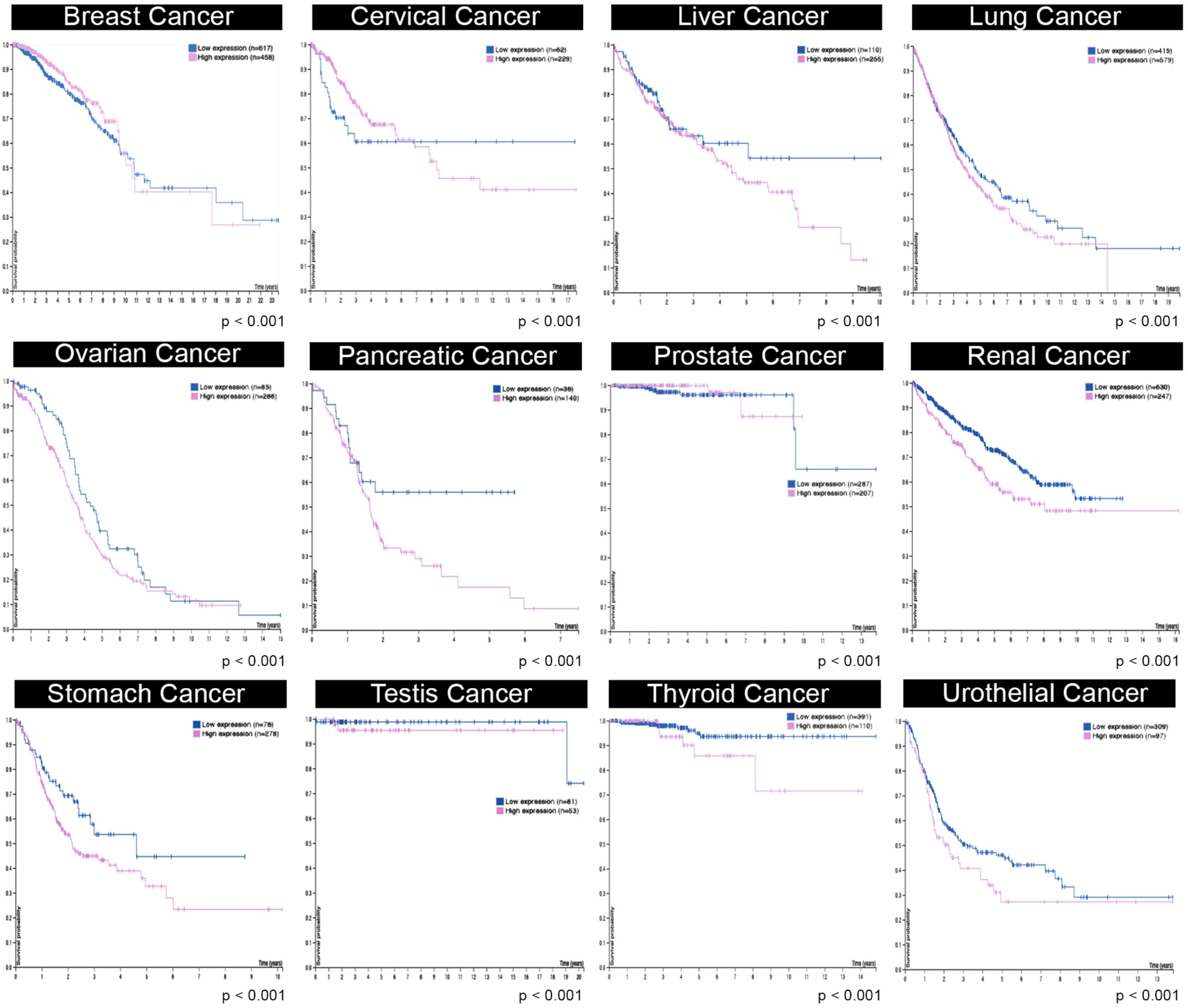

Supplementary Fig. 6 | Analyses of the TCGA data, protein atlas and Kaplan-Meier survival analysis for D2 in different human malignancies. Kaplan-Meier survival plots showing the Overall Survival (OS) in distinct tumor types in which patients were stratified in high (pink) and low (blue) based on *DIO2* expression (www.proteinatlas.org/ENSG00000211448). p-values were determined by log-rank tests and the number of patients in each group is provided in the figure. Source data are provided as a Source Data file.

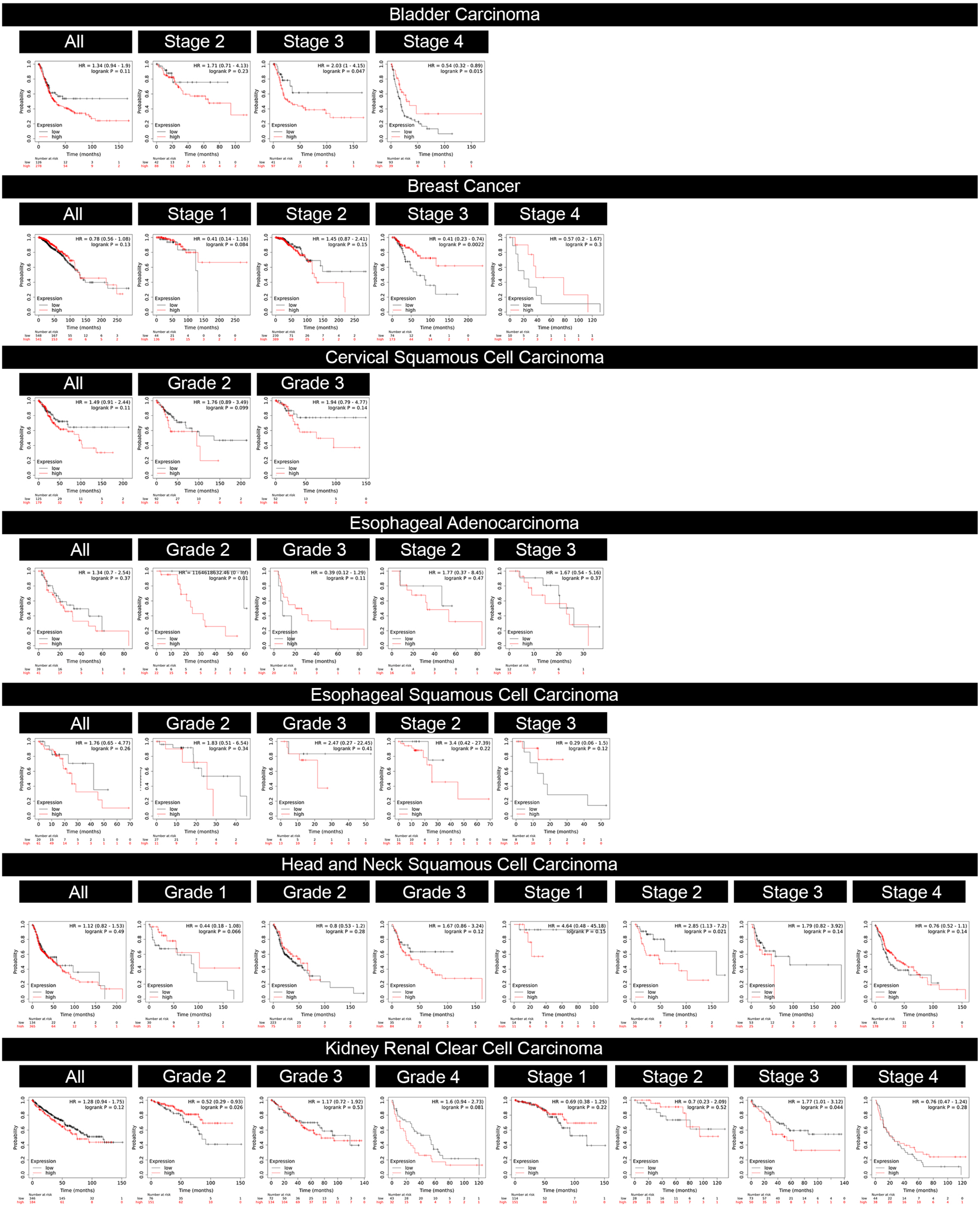

Supplementary Fig. 7 | Correlation of *DIO2* expression with different tumor types. Kaplan-Meier survival plots showing the Overall Survival (OS) in distinct tumor types in which patients were stratified in high (red) and low (black) based on *DIO2*/*TP53* expression ratio and divided, where possible, for tumor stage and grade using the KM-Plotter data set (doi.org/10.1038/s41598-021-84787-5). p-values were determined by log-rank tests and the number of patients in each group is provided in the figure.

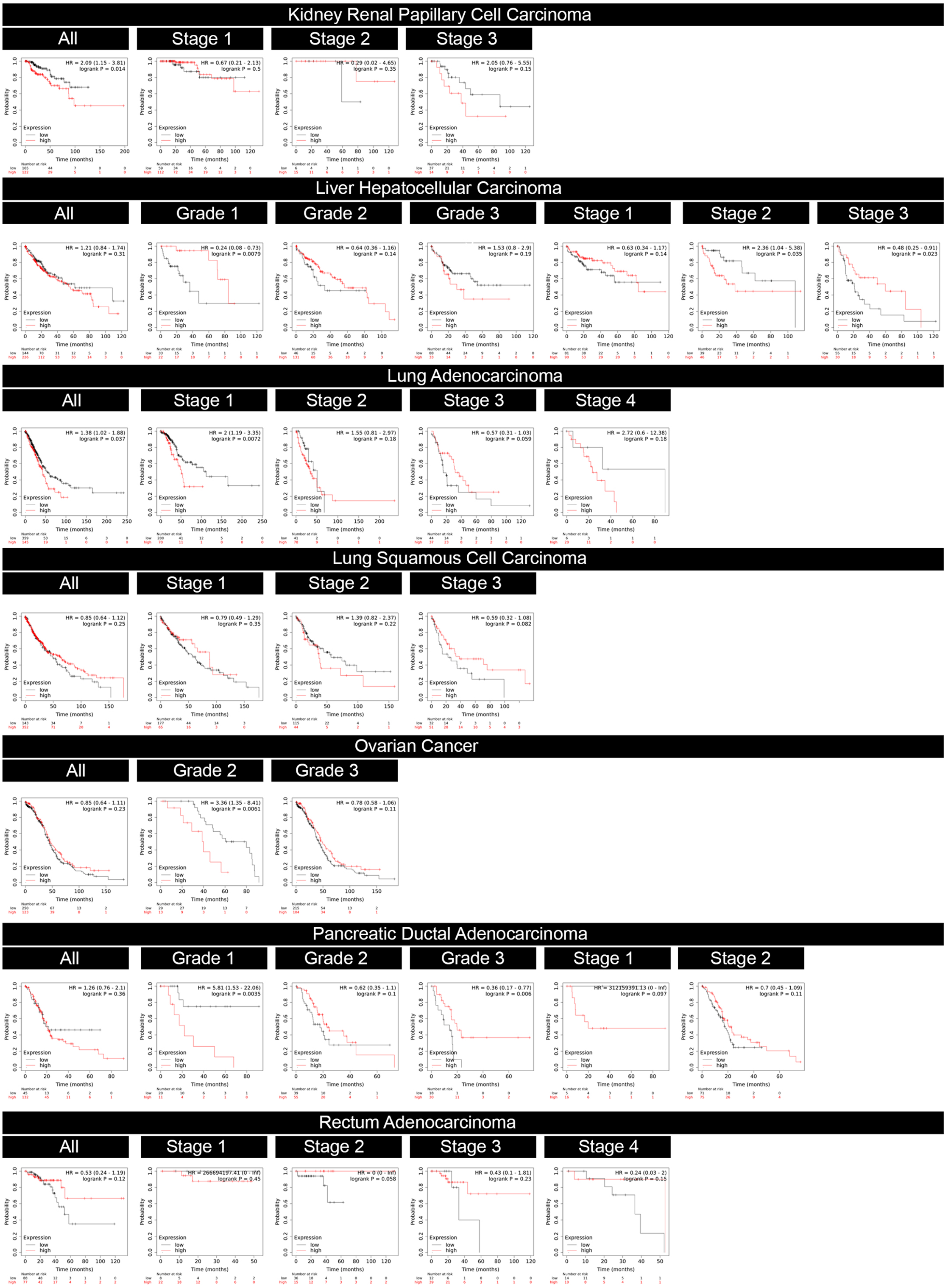

Supplementary Fig. 8 | Correlation of *DIO2* expression with different tumor types. Kaplan-Meier survival plots showing the Overall Survival (OS) in distinct tumor types in which patients were stratified in high (red) and low (black) based on *DIO2*/*TP53* expression ratio and divided, where possible, for tumor stage and grade using the KM-Plotter data set (doi.org/10.1038/s41598-021-84787-5). p-values were determined by log-rank tests and the number of patients in each group is provided in the figure.

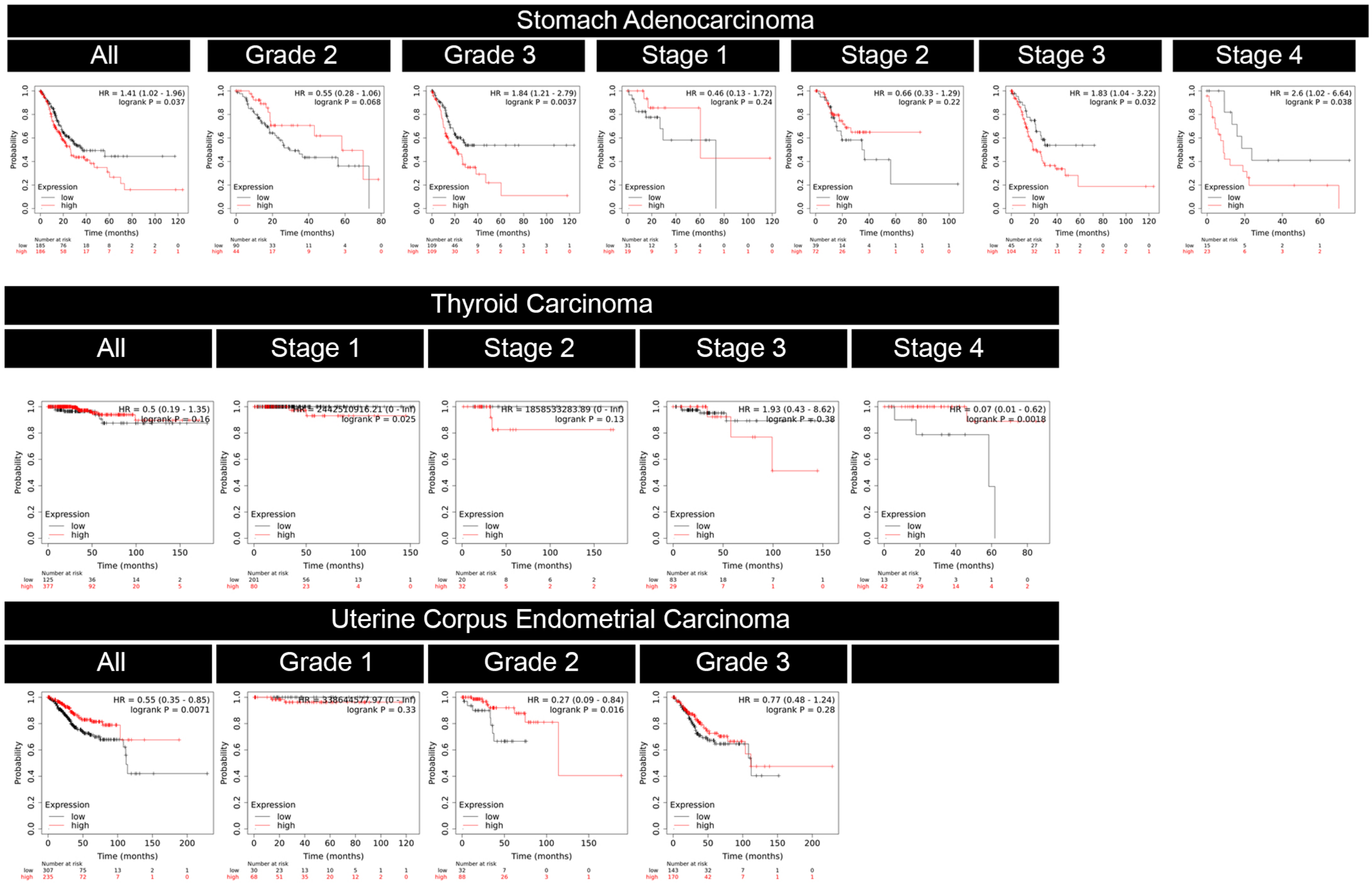

Supplementary Fig. 9 | Correlation of *DIO2* expression with different tumor types. Kaplan-Meier survival plots showing the Overall Survival (OS) in distinct tumor types in which patients were stratified in high (red) and low (black) based on *DIO2*/*TP53* expression ratio and divided, where possible, for tumor stage and grade using the KM-Plotter data set (doi.org/10.1038/s41598-021-84787-5). p-values were determined by log-rank tests and the number of patients in each group is provided in the figure.

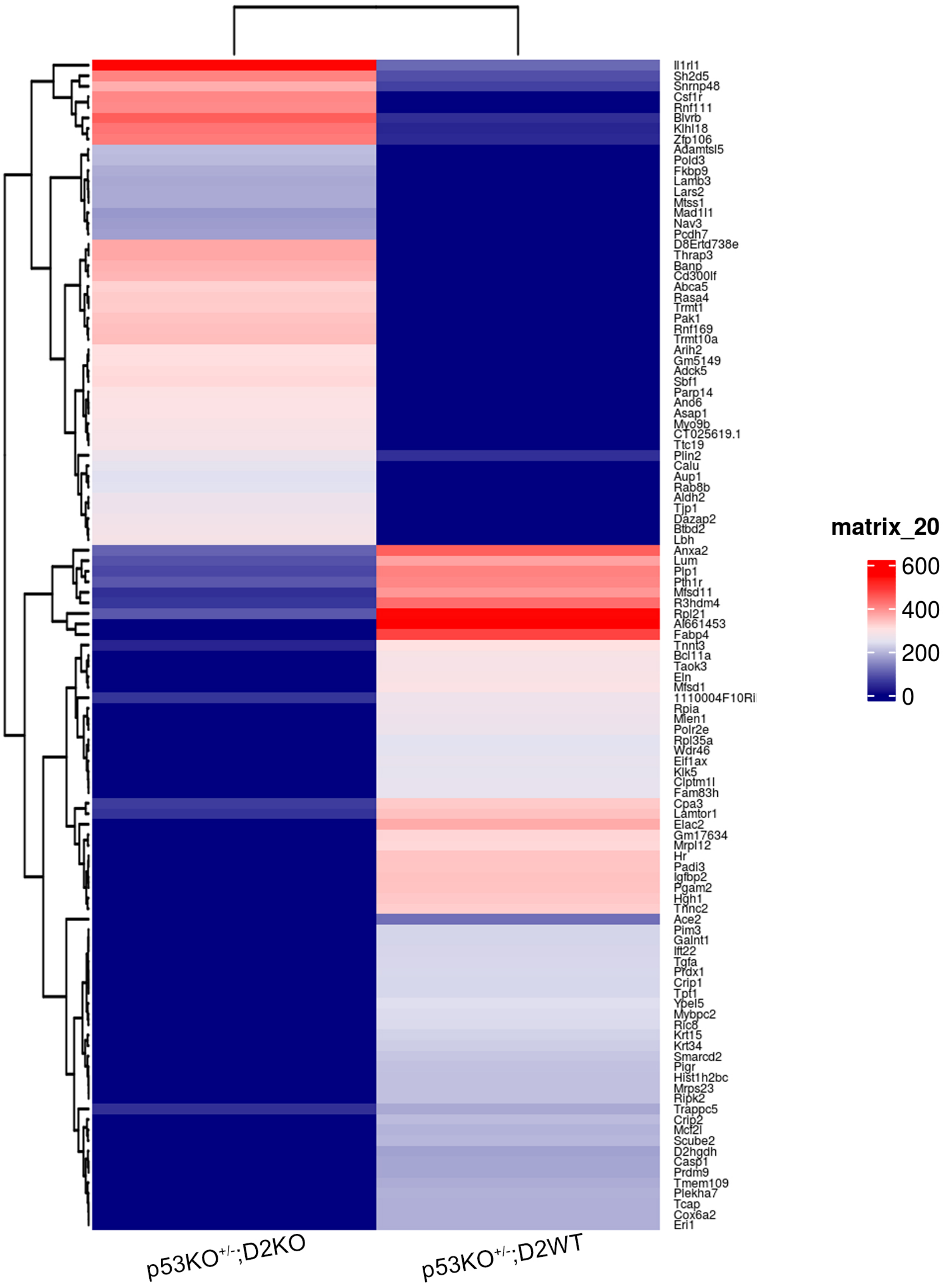

Supplementary Fig. 10 | D2 inhibition fosters the DNA damage repair mechanisms in skin cancer. Expression heatmap of 111 Differentially Expressed Genes (DEGs) that were expressed across i) p53KO$^{+/-}$;D2WT and ii) p53KO$^{+/-}$;D2KO mice.

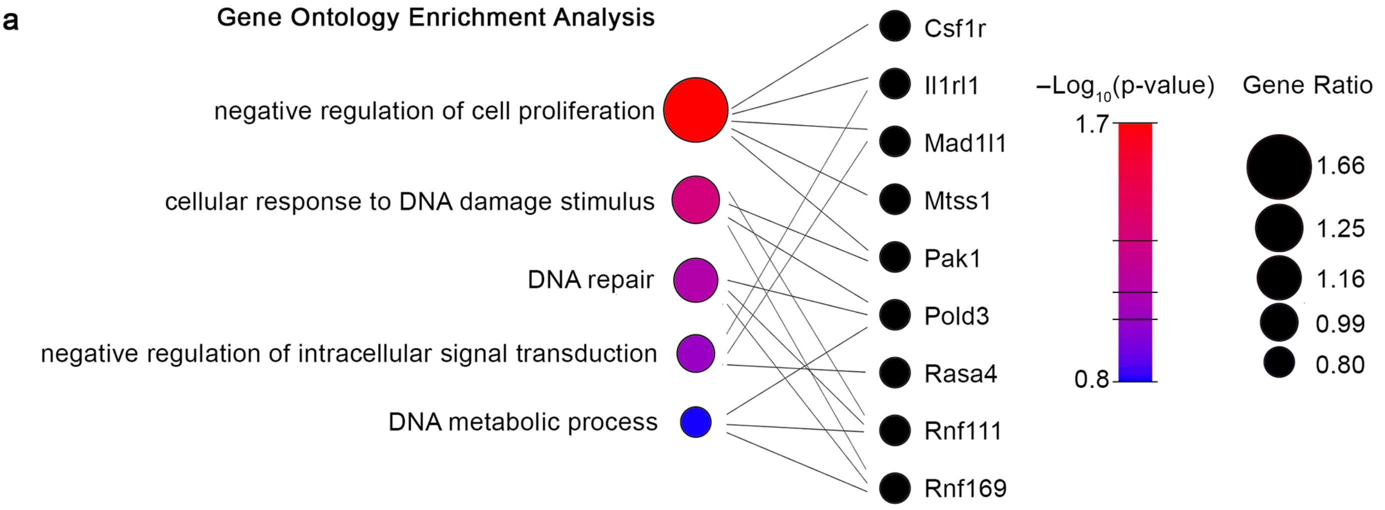

b

Genes	Term
Pak1	regulation of cell cycle (GO:0051726)<br>regulation of programmed cell death (GO:0043067)<br>regulation of cell proliferation (GO:0042127)
Mad1l1	mitotic spindle assembly checkpoint (GO:0007094)<br>mitotic spindle checkpoint (GO:0071174)<br>regulation of cell cycle process (GO:0010564)
Pold3	nucleotide-excision repair (GO:0006289)<br>nucleotide-excision repair, DNA gap filling (GO:0006297)<br>mismatch repair (GO:0006298)<br>DNA biosynthetic process (GO:0071897)<br>nucleotide-excision repair, DNA incision (GO:0033683)<br>DNA synthesis involved in DNA repair (GO:0000731)<br>DNA repair (GO:0006281)
Rnf169	negative regulation of DNA repair (GO:0045738)<br>negative regulation of double-strand break repair (GO:2000780)<br>regulation of double-strand break repair (GO:2000779)<br>cellular response to DNA damage stimulus (GO:0006974)
Rnf111	nucleotide-excision repair (GO:0006289)

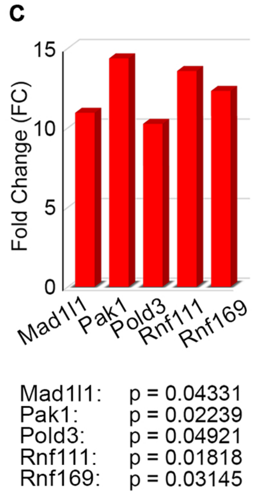

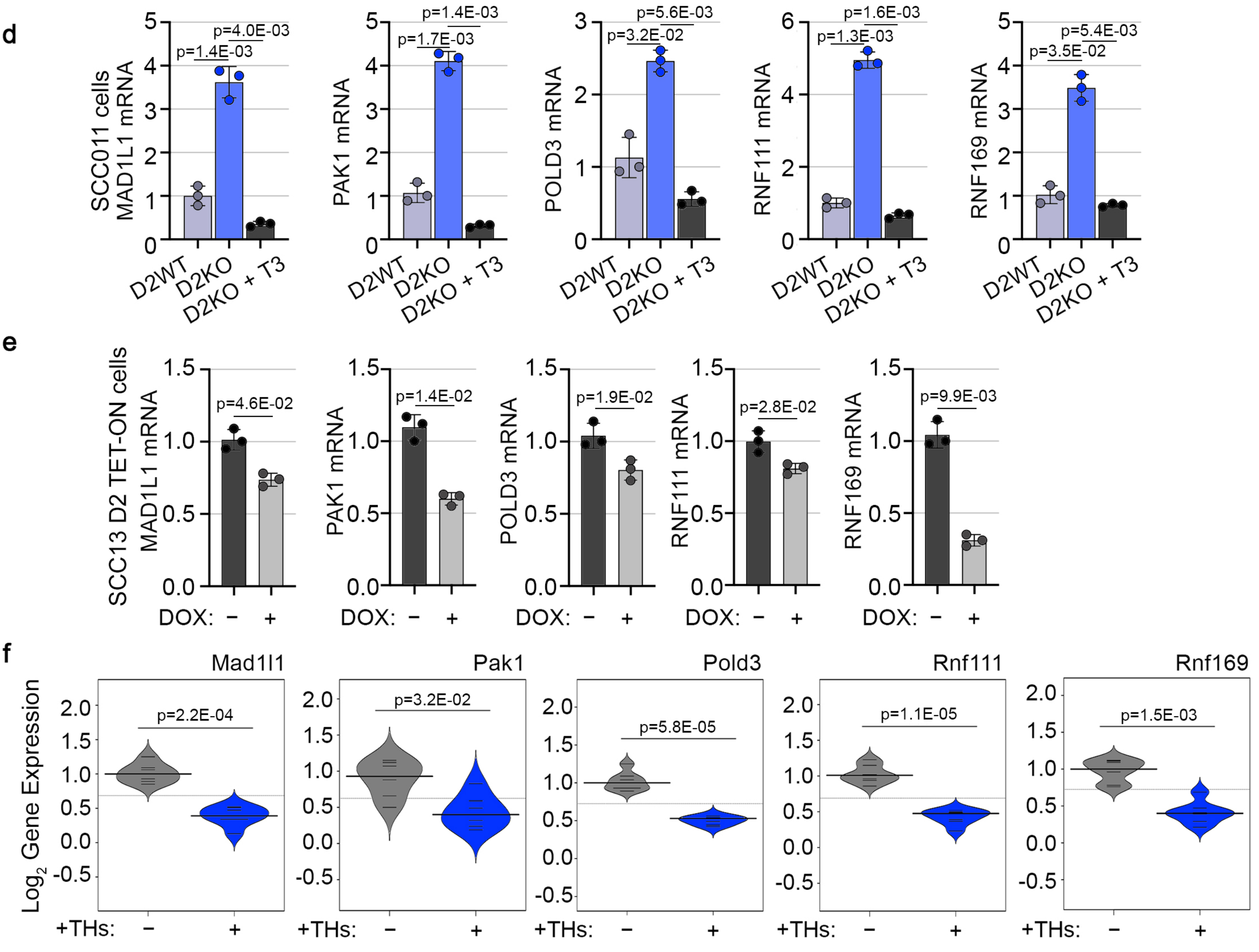

Supplementary Fig. 11 | TH signaling impairs the DNA damage repair mechanisms in skin tumorigenesis. a, Gene Ontology (GO) Enrichment Analysis of Biological Processes (BPs) and functional cluster analysis were analyzed by Metascape Gene Annotation & Analysis Resource

(https://metascape.org/gp/index). GO terms were extracted analyzing all the 111 identified DEGs (65 of them negatively regulated for value of Log_2 Fold-Change (FC) $\leq - 1.5$ and 46 positively regulated for value of Log_2 Fold-Change (FC) $\geq - 1.5$, both with $p < 0.05$) across i) p53KO$^{+/-}$;D2WT and ii) p53KO$^{+/-}$;D2KO mice. **b**, Gene Ontology (GO) enrichment of the top 5 selected DEGs, namely *Mad1l1*, *Pak1*, *Pold3*, *Rnf111* and *Rnf169*, according to the molecular function category. **c**, Fold Change (FC) expression of the top 5 DEGs from RNA-seq analysis of SCC tumors from dorsal skin of i) p53KO$^{+/-}$;D2WT and ii) p53KO$^{+/-}$;D2KO mice. **d**, mRNA expression of *MAD1L1*, *PAK1*, *POLD3*, *RNF111* and *RNF169* was measured by Real-Time PCR in D2WT *versus* D2KO SCC011 cells, treated or not with T3 (30.0 nM/24 hours) (n = 3 independent experiments). **e**, mRNA expression of *MAD1L1*, *PAK1*, *POLD3*, *RNF111* and *RNF169* was measured by Real-Time PCR in SCC13 Tet-ON-D2 cells treated or not with doxycycline (DOX, 2 µg/mL) for 24 hours (n = 3 independent experiments). **f**, Bean Plots represent the Log_2 gene expression of the top 5 DEGs measured by Real-Time PCR in SCC tumors from dorsal skin of p53WT;D2WT and p53WT;D2KO mice, treated or not with THs in drinking water (n = 7 mice/group). All the results are shown as means ± SD from at least 3 separate experiments. p-values were determined by two-tailed Student's t-test. Source data are provided as a Source Data file.

a (i)

AC	ID	Score	Loc.	Str.	Consensus Sequence	Signal Sequence
M00239	V$T3R_01	0.730078	74	(-)	SNNTRAGGTCACGSNN	CTTCTTGGCCACACAG
M00239	V$T3R_01	0.794814	98	(+)	SNNTRAGGTCACGSNN	AAGGAAGGGCACTGTG
M00239	V$T3R_01	0.788756	298	(+)	SNNTRAGGTCACGSNN	AACTAAGTTCAGGGGC
M00239	V$T3R_01	0.736513	460	(-)	SNNTRAGGTCACGSNN	TGCCTTGACGGGACTT
M00239	V$T3R_01	0.748060	526	(+)	SNNTRAGGTCACGSNN	CTCTGAGAGCACCACG
M00239	V$T3R_01	0.832671	594	(+)	SNNTRAGGTCACGSNN	GGGTGAGGCCACTCAT
M00239	V$T3R_01	0.763013	652	(+)	SNNTRAGGTCACGSNN	GCTTGAAGTCAGGAGT
M00239	V$T3R_01	0.739920	1470	(+)	SNNTRAGGTCACGSNN	CAGGATGGTCTCGATC
M00239	V$T3R_01	0.766799	1840	(+)	SNNTRAGGTCACGSNN	GACAAAGGTTACCATA
M00239	V$T3R_01	0.890403	1865	(-)	SNNTRAGGTCACGSNN	GGGCCTGACCTCACCC
M00239	V$T3R_01	0.780806	1867	(+)	SNNTRAGGTCACGSNN	GCCTGACCTCACCCCA
M00239	V$T3R_01	0.739920	1888	(-)	SNNTRAGGTCACGSNN	TGCTGTGCCCTGGCTT

(ii)

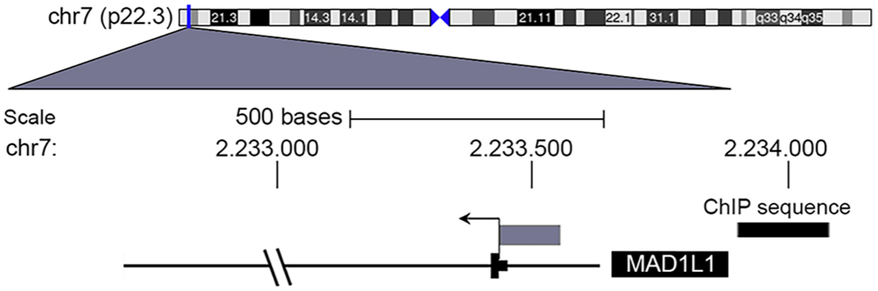

b (i)

AC	ID	Score	Loc.	Str.	Consensus Sequence	Signal Sequence
M00239	V$T3R_01	0.801439	129	(-)	SNNTRAGGTCACGSNN	ACTTCTGACCTCAGGT
M00239	V$T3R_01	0.743706	153	(-)	SNNTRAGGTCACGSNN	TGCCTTGGCCTCCCAA
M00239	V$T3R_01	0.730645	196	(-)	SNNTRAGGTCACGSNN	GCGCCTGGCCTTATTA
M00239	V$T3R_01	0.731024	491	(+)	SNNTRAGGTCACGSNN	GCTTGAAGACATGAAG
M00239	V$T3R_01	0.768124	595	(+)	SNNTRAGGTCACGSNN	CAGTCAGGTAATAAAG
M00239	V$T3R_01	0.752792	623	(-)	SNNTRAGGTCACGSNN	GAGTTTGAACTTTATC
M00239	V$T3R_01	0.749574	670	(+)	SNNTRAGGTCACGSNN	ACGGGAGGACATCATC
M00239	V$T3R_01	0.761121	1262	(+)	SNNTRAGGTCACGSNN	ATCTGAGCTCAGGAGA
M00239	V$T3R_01	0.738974	1349	(+)	SNNTRAGGTCACGSNN	ATGTCAGTTCACATTA
M00239	V$T3R_01	0.777021	1448	(-)	SNNTRAGGTCACGSNN	GGCTGTGAGATCACAC
M00239	V$T3R_01	0.857657	1450	(+)	SNNTRAGGTCACGSNN	CTGTGAGATCACACGA
M00239	V$T3R_01	0.738785	1494	(-)	SNNTRAGGTCACGSNN	GACAACGAGCTCATAG
M00239	V$T3R_01	0.734242	1496	(+)	SNNTRAGGTCACGSNN	CAACGAGCTCATAGAT
M00239	V$T3R_01	0.757903	1668	(-)	SNNTRAGGTCACGSNN	AACAGTGACGCGACGC
M00239	V$T3R_01	0.831724	1681	(-)	SNNTRAGGTCACGSNN	CGCGATGACGTCACGC
M00239	V$T3R_01	0.852735	1683	(+)	SNNTRAGGTCACGSNN	CGATGACGTCACGCAG
M00239	V$T3R_01	0.829642	1719	(+)	SNNTRAGGTCACGSNN	CTCTGGCGTCACGCGC
M00239	V$T3R_01	0.736324	1733	(-)	SNNTRAGGTCACGSNN	GCCGATGACGTAATCC
M00239	V$T3R_01	0.742949	1735	(+)	SNNTRAGGTCACGSNN	CGATGACGTAATCCCA
M00239	V$T3R_01	0.760742	1774	(-)	SNNTRAGGTCACGSNN	GGGTTTGGCCTCGCTC
M00239	V$T3R_01	0.736135	1836	(+)	SNNTRAGGTCACGSNN	CGGGGCGGGCACGGCT
M00239	V$T3R_01	0.748060	1975	(+)	SNNTRAGGTCACGSNN	CTAGGCGGTCAGGCCT

(ii)

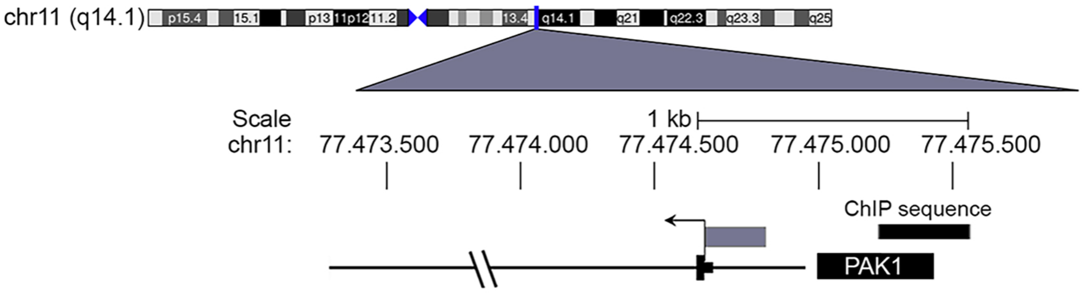

Supplementary Fig. 12 | Positions of Thyroid Hormone Receptor binding sites within *MAD1L1* and *PAK1* promoter genes. a, **(i)** Consensus sequence analysis of the 12 different putative consensus motifs for Thyroid Hormone Receptor (ID V$T3R_01, AC M00239) within the *MAD1L1* promoter region. **(ii)** The mammalian structure and position from Genome Browser are indicated for the *MAD1L1* promoter. The black box represents the sequence analyzed by ChIP. **b**, **(i)** Consensus sequence analysis of the 22 different putative consensus motifs for Thyroid Hormone Receptor (ID V$T3R_01, AC M00239) within the *PAK1* promoter region. **(ii)** The mammalian structure and position from Genome Browser are indicated for the *PAK1* promoter. The black box represents the sequence analyzed by ChIP.

a (i)

AC	ID	Score	Loc.	Str.	Consensus Sequence	Signal Sequence
M00239	V$T3R_01	0.761310	118	(-)	SNNTRAGGTCACGSNN	AACTGGGACCACAGAC
M00239	V$T3R_01	0.743706	249	(-)	SNNTRAGGTCACGSNN	TGCCTTGGCCTCCCAA
M00239	V$T3R_01	0.776831	336	(+)	SNNTRAGGTCACGSNN	GAGGGGGGTCTCACTC
M00239	V$T3R_01	0.816392	381	(+)	SNNTRAGGTCACGSNN	ATCTCAGCTCACCGCA
M00239	V$T3R_01	0.830778	539	(-)	SNNTRAGGTCACGSNN	ACTCCTGACCTCAGGT
M00239	V$T3R_01	0.755821	670	(-)	SNNTRAGGTCACGSNN	TGGCATGATCTCGGCT
M00239	V$T3R_01	0.759417	677	(+)	SNNTRAGGTCACGSNN	ATCTCGGCTCACTGCA
M00239	V$T3R_01	0.830778	835	(-)	SNNTRAGGTCACGSNN	ACTCCTGACCTCAGGT
M00239	V$T3R_01	0.755631	842	(+)	SNNTRAGGTCACGSNN	ACCTCAGGTGATTCAT
M00239	V$T3R_01	0.746356	1157	(-)	SNNTRAGGTCACGSNN	AAGAATTACCTAAGTT
M00239	V$T3R_01	0.740867	1164	(+)	SNNTRAGGTCACGSNN	ACCTAAGTTTACACAA
M00239	V$T3R_01	0.770396	1714	(-)	SNNTRAGGTCACGSNN	TTGAGAGACCATATTC
M00239	V$T3R_01	0.731970	1857	(+)	SNNTRAGGTCACGSNN	CCAGGAGGCCCCGCAC
M00239	V$T3R_01	0.739542	1952	(-)	SNNTRAGGTCACGSNN	GTGCGTCGCCTCCGTT
M00239	V$T3R_01	0.742381	1980	(-)	SNNTRAGGTCACGSNN	ACCCGGGACCTGGGAG

(ii)

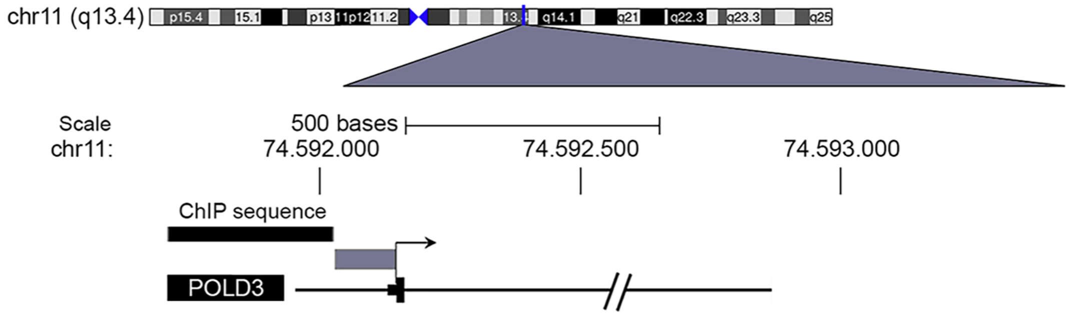

b (i)

AC	ID	Score	Loc.	Str.	Consensus Sequence	Signal Sequence
M00239	V$T3R_01	0.751656	295	(+)	SNNTRAGGTCACGSNN	AACACAGGTCAAAAGC
M00239	V$T3R_01	0.731970	367	(+)	SNNTRAGGTCACGSNN	AGTTAAAATCAAGGGA
M00239	V$T3R_01	0.750331	436	(+)	SNNTRAGGTCACGSNN	GATTGAGGAAAATCCT
M00239	V$T3R_01	0.770017	685	(+)	SNNTRAGGTCACGSNN	AGCTGGGGTTACAGGC
M00239	V$T3R_01	0.782889	779	(-)	SNNTRAGGTCACGSNN	TCTCCTGACCTCATGA
M00239	V$T3R_01	0.731024	801	(-)	SNNTRAGGTCACGSNN	CGCCTTGGCCTCCAAA
M00239	V$T3R_01	0.743328	1075	(-)	SNNTRAGGTCACGSNN	ACTCCTGATCTCAGGT
M00239	V$T3R_01	0.747113	1213	(+)	SNNTRAGGTCACGSNN	AGATGAGTTGATGGCA
M00239	V$T3R_01	0.751656	1569	(-)	SNNTRAGGTCACGSNN	ACTTTTGATCTGATTC
M00239	V$T3R_01	0.738974	1605	(+)	SNNTRAGGTCACGSNN	CAGTGAGGTGCCGTGA
M00239	V$T3R_01	0.819231	1613	(-)	SNNTRAGGTCACGSNN	TGCCGTGATCTTTATG

(ii)

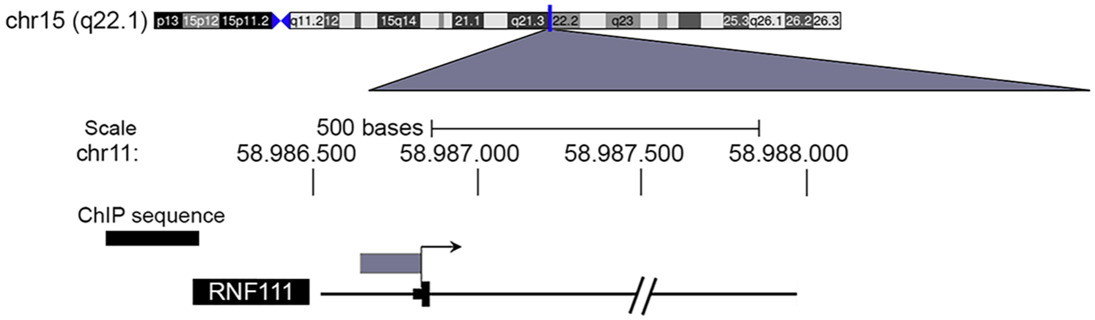

Supplementary Fig. 13 | Positions of Thyroid Hormone Receptor binding sites within *POLD3* and *RNF111* promoter genes. a, **(i)** Consensus sequence analysis of the 15 different putative consensus motifs for Thyroid Hormone Receptor (ID V$T3R_01, AC M00239) within the *POLD3* promoter region. **(ii)** The mammalian structure and position from Genome Browser are indicated for the *POLD3* promoter. The black box represents the sequence analyzed by ChIP. **b**, **(i)** Consensus sequence analysis of the 11 different putative consensus motifs for Thyroid Hormone Receptor (ID V$T3R_01, AC M00239) within the *RNF111* promoter region. **(ii)** The mammalian structure and position from Genome Browser are indicated for the *RNF111* promoter. The black box represents the sequence analyzed by ChIP.

a (i)

AC	ID	Score	Loc.	Str.	Consensus Sequence	Signal Sequence
M00239	V$T3R_01	0.743517	206	(-)	SNNTRAGGTCACGSNN	ATCCATCACCTTTTTT
M00239	V$T3R_01	0.747113	383	(-)	SNNTRAGGTCACGSNN	AGTGGTGCCATCACGG
M00239	V$T3R_01	0.837403	407	(-)	SNNTRAGGTCACGSNN	AGCCTTAACCTCATGG
M00239	V$T3R_01	0.758471	409	(+)	SNNTRAGGTCACGSNN	CCTTAACCTCATGGAC
M00239	V$T3R_01	0.813742	425	(-)	SNNTRAGGTCACGSNN	TCCAGTGATCTTCCTG
M00239	V$T3R_01	0.763013	540	(-)	SNNTRAGGTCACGSNN	ACTCCTGAGCTCAAGC
M00239	V$T3R_01	0.773992	542	(+)	SNNTRAGGTCACGSNN	TCCTGAGCTCAAGCGA
M00239	V$T3R_01	0.792921	783	(+)	SNNTRAGGTCACGSNN	GAGAGGGGTCATTCTT
M00239	V$T3R_01	0.774938	1104	(+)	SNNTRAGGTCACGSNN	GAAGAAGTTCAAGATG
M00239	V$T3R_01	0.849707	1208	(-)	SNNTRAGGTCACGSNN	GACAGTGACATTAGTT
M00239	V$T3R_01	0.752981	1377	(+)	SNNTRAGGTCACGSNN	GACTGAGGCCTAAGCC
M00239	V$T3R_01	0.760931	1616	(-)	SNNTRAGGTCACGSNN	AGGAGAGAGCTCAGGC
M00239	V$T3R_01	0.741813	1618	(+)	SNNTRAGGTCACGSNN	GAGAGAGCTCAGGCCC
M00239	V$T3R_01	0.731592	1831	(-)	SNNTRAGGTCACGSNN	GCTGATGACCGCATAA
M00239	V$T3R_01	0.738785	1889	(+)	SNNTRAGGTCACGSNN	CAATCAGGTCTGAGGA
M00239	V$T3R_01	0.740488	1896	(+)	SNNTRAGGTCACGSNN	GTCTGAGGAGAACCCT
M00239	V$T3R_01	0.805414	1912	(+)	SNNTRAGGTCACGSNN	CTTTGGGCTCACAGAA

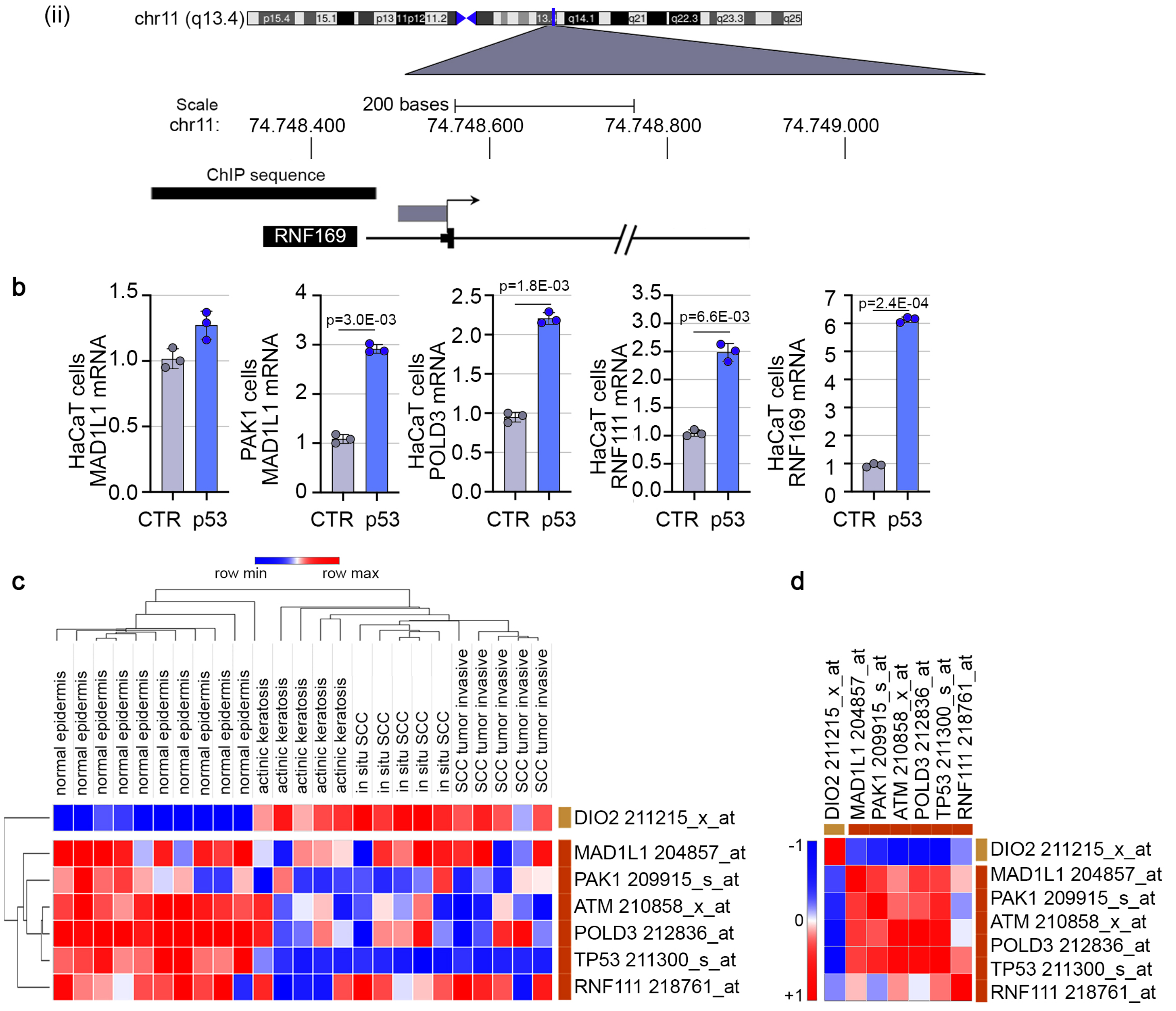

Supplementary Fig. 14 | Positions of Thyroid Hormone Receptor binding sites within *RNF169* promoter gene. **a**, **(i)** Consensus sequence analysis of the 17 different putative consensus motifs for Thyroid Hormone Receptor (ID V$T3R_01, AC M00239) within the *RNF169* promoter region. **(ii)** The mammalian structure and position from Genome Browser are indicated for the *RNF169* promoter. The black box represents the sequence analyzed by ChIP. **b**, *MAD1L1*, *PAK1*, *POLD3*, *RNF111* and *RNF169* mRNA expression was measured by Real-Time PCR in HaCaT cells transiently transfected with a p53-expressing vector or a control empty plasmid (CTR). All the results are shown

as means ± SD from at least 3 separate experiments. p-values were determined by two-tailed Student's t-test. **c**, Expression heatmap of *DIO2*, *MAD1L1*, *PAK1*, *ATM*, *POLD3*, *TP53* and *RNF111* genes, differentially expressed across normal epidermis, actinic keratosis, in situ and invasive SCC samples from GSE42677. **d**, Pearson's correlation analysis was performed for the same dataset samples as in a, by using Morpheus heatmap visualization software (Pearson's correlation coefficient, ρ: $\rho_{DIO2} = -0.98$; $\rho_{MAD1L1} = 0.51$; $\rho_{PAK1} = 0.54$; $\rho_{ATM} = 0.73$; $\rho_{POLD3} = 0.80$; $\rho_{TP53} = 0.93$; $RNF111 = 0.23$). Source data are provided as a Source Data file.

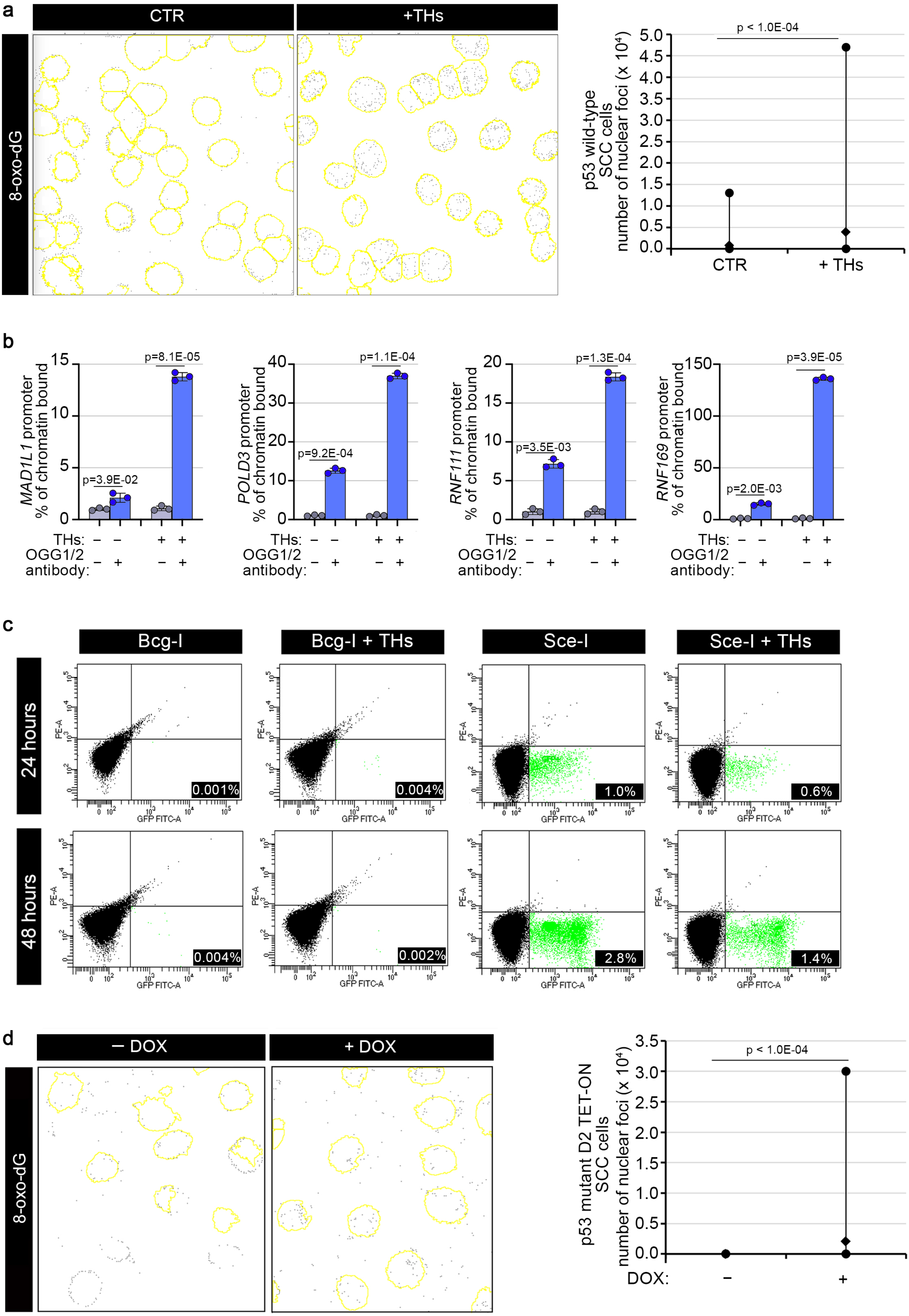

Supplementary Fig. 15 | THs treatment induce a significant accumulation of nuclear 8-oxo-dG signal and affect DNA repair mechanisms. a, Quantification analysis of the 8-oxo-dG/DAPI co-localization signal in SCC011 cells treated or not with THs (T3, 30.0 nM/24 hours + T4, 30.0 nM/24

hours). p-value was determined by Mann–Whitney test. **b**, ChIP of the 8-oxoguanine DNA glycosylase 1, OGG1/2, binding to the *MAD1L1*, *POLD3*, *RNF111* and *RNF169* promoter was performed in SCC011 cells treated or not with THs (T3, 30.0 nM/24 hours + T4, 30.0 nM/24 hours). Graph shows the Real-Time PCR results with % chromatin bound as indicated. All the results are shown as means ± SD from at least 3 separate experiments. p-values were determined by two-tailed Student's t-test. **c**, Cytofluorimetric analysis of HeLa-DRGFP cells, treated or not with THs (T3, 30.0 nM + T4, 30.0 nM) for 24 and 48 hours, undergoing repair by homologous recombination. The panels show the distribution of GFP^{+} cells after homologous recombination. **d**, Quantification analysis of the 8-oxo-dG/DAPI co-localization signal in SCC13 Tet-ON-D2 cells treated or not with doxycycline (DOX, 2 µg/mL) for 24 hours. p-value was determined by Mann–Whitney test. Source data are provided as a Source Data file.

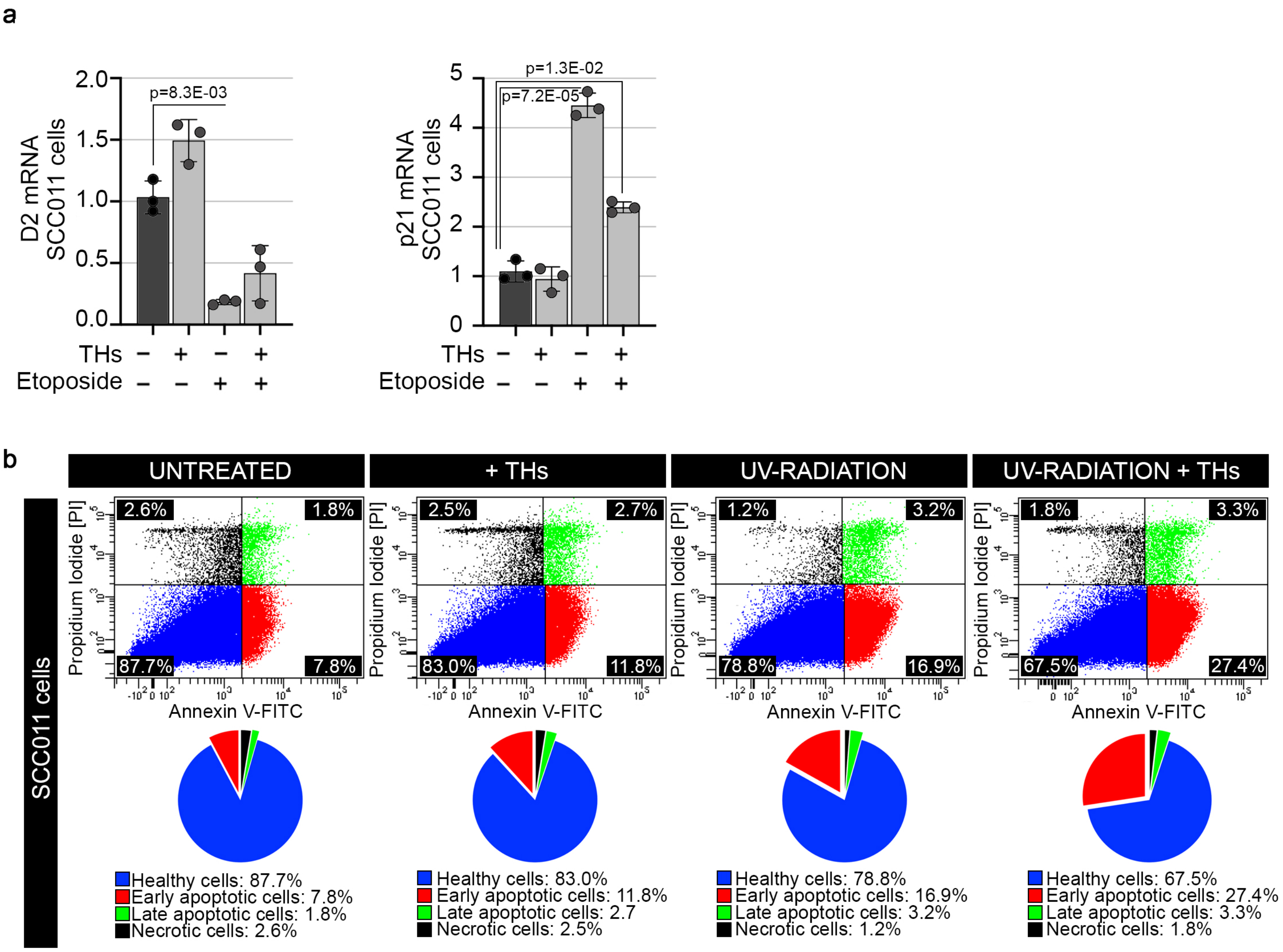

Supplementary Fig. 16 | Enhancement of TH signaling increases DNA damage. a, mRNA expression of *D2* and *p21* was measured by Real-Time PCR in SCC011, treated or not with THs (T3, 30.0 nM + T4, 30.0 nM) for 24 hours, alone and in combination with etoposide (50.0 µM/30 minutes) (n = 3 independent experiments). All the results are shown as means ± SD from at least 3 separate experiments. p-values were determined by two-tailed Student's t-test. **b**, Flow cytometric analysis for monitoring the effect of TH-induced apoptosis in SCC011 cells, exposed or not to UV-C radiation. Pie charts represent percentages of Viable (V), Early (E) and Late (L) apoptotic, and Necrotic (N) cells measured by Annexin V-FITC/PI costaining. Graphs represent an average of 3 separate experiments. Source data are provided as a Source Data file.

Figure 7a

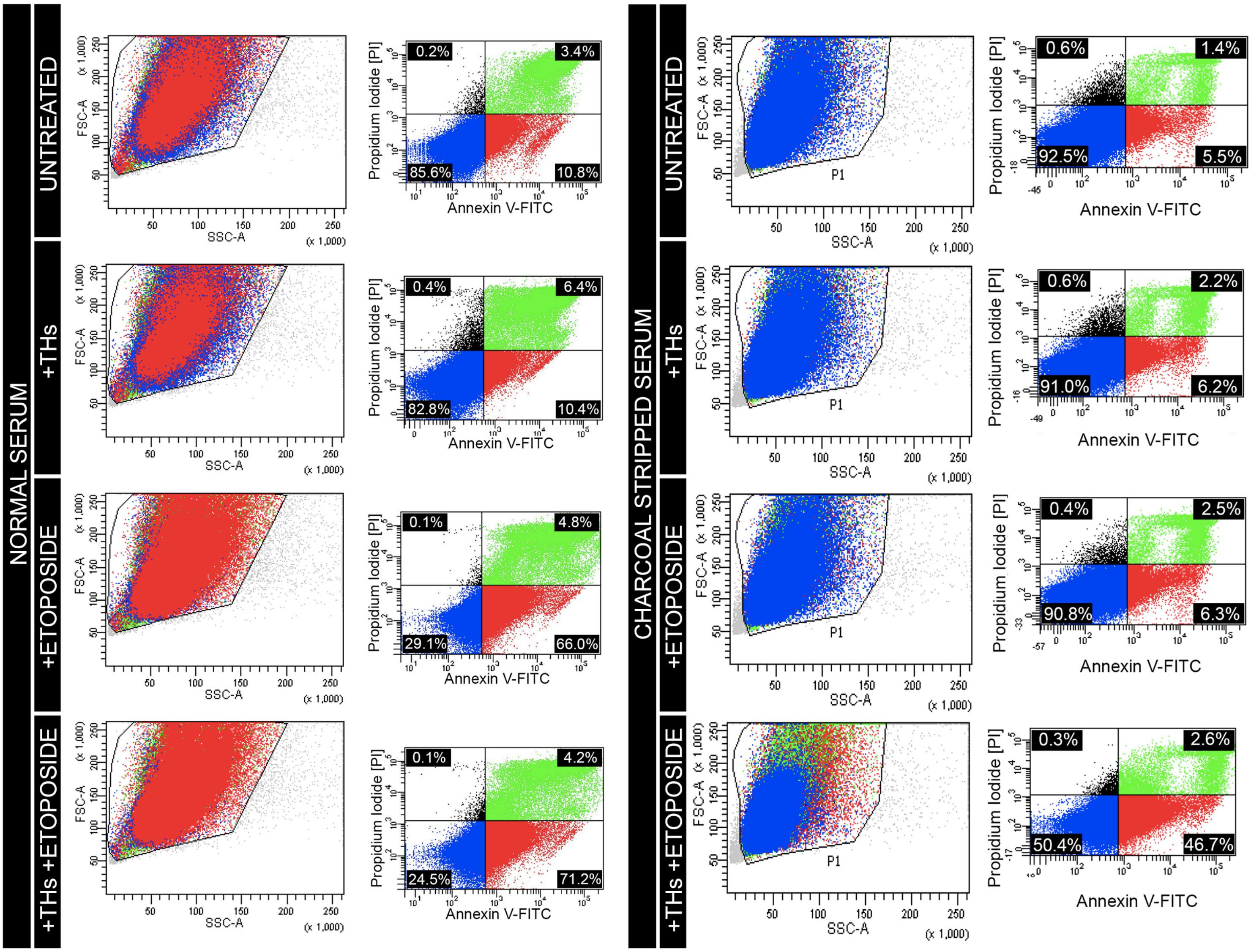

Figure 7c

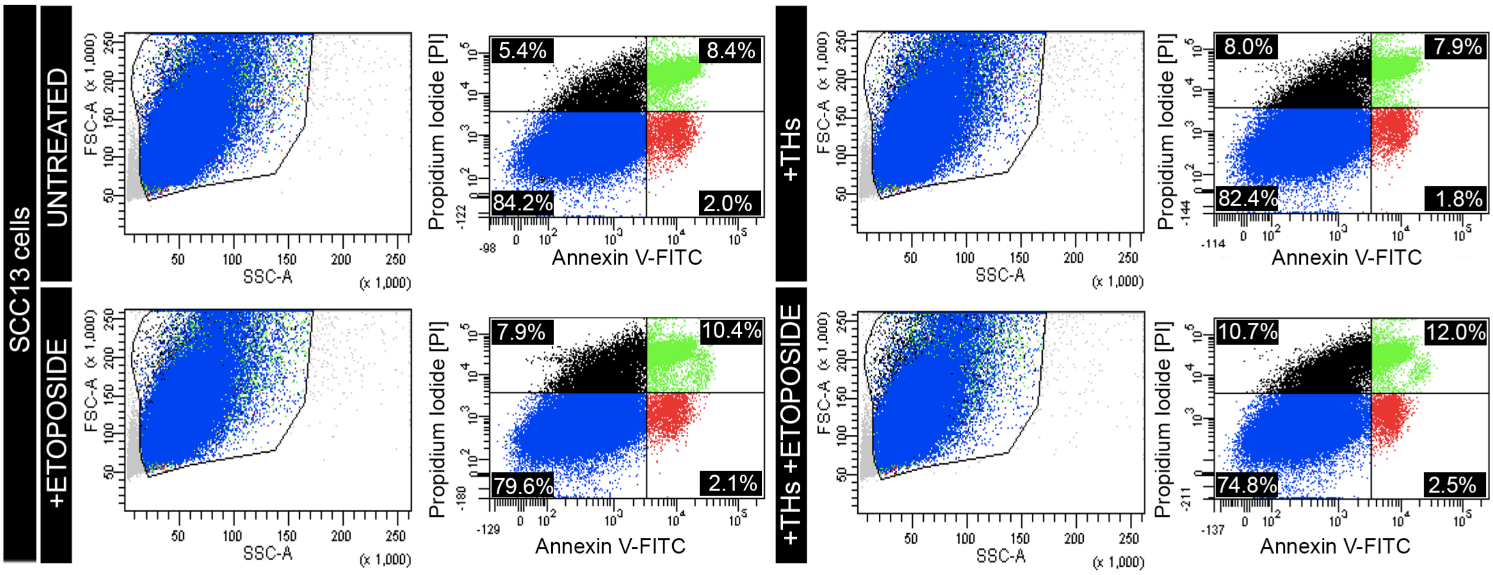

Supplementary Fig. 17 | Graphical representation for all FACS sequential gating/sorting strategies.

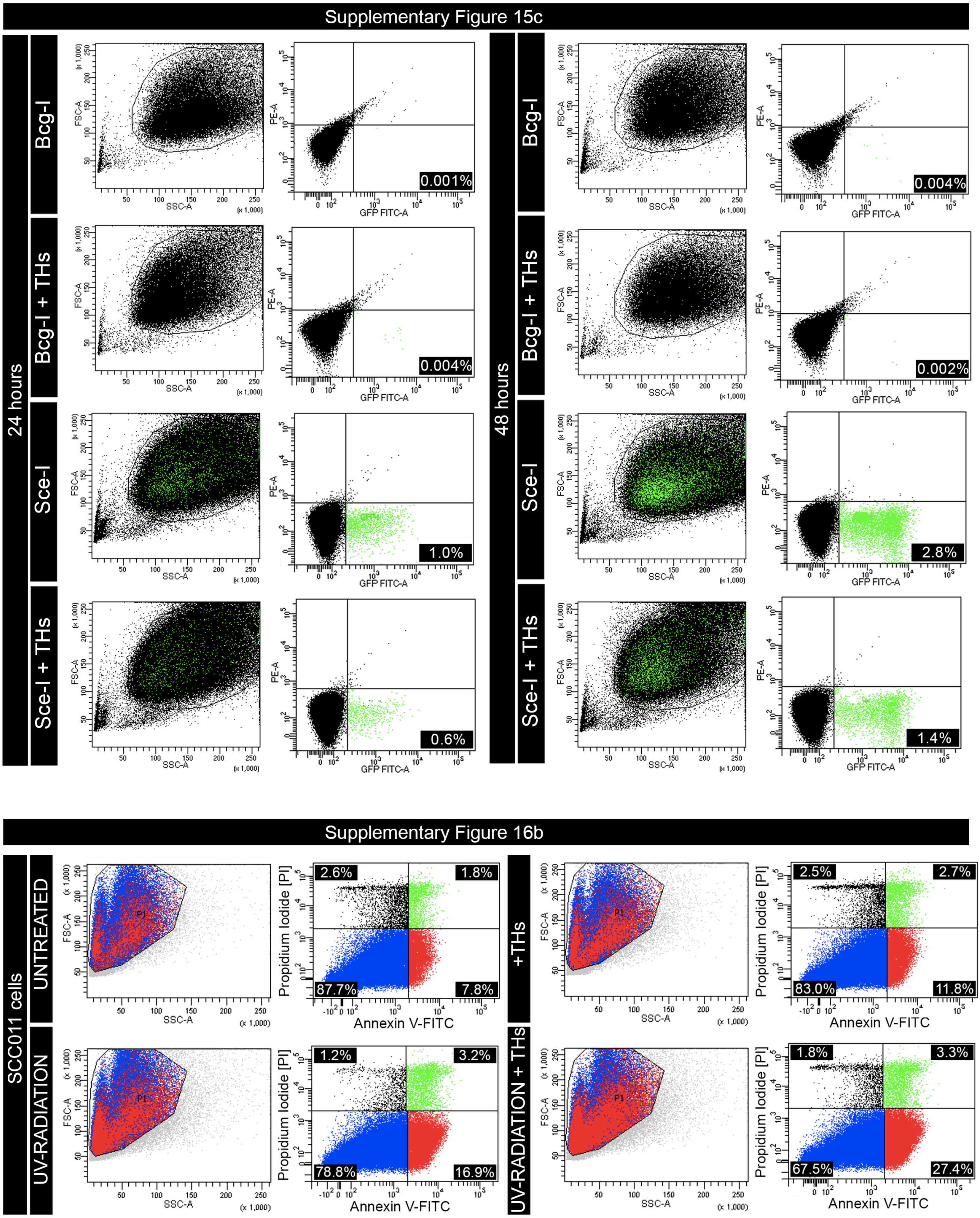

Supplementary Fig. 18 | Graphical representation for all FACS sequential gating/sorting strategies.

Figure 2h

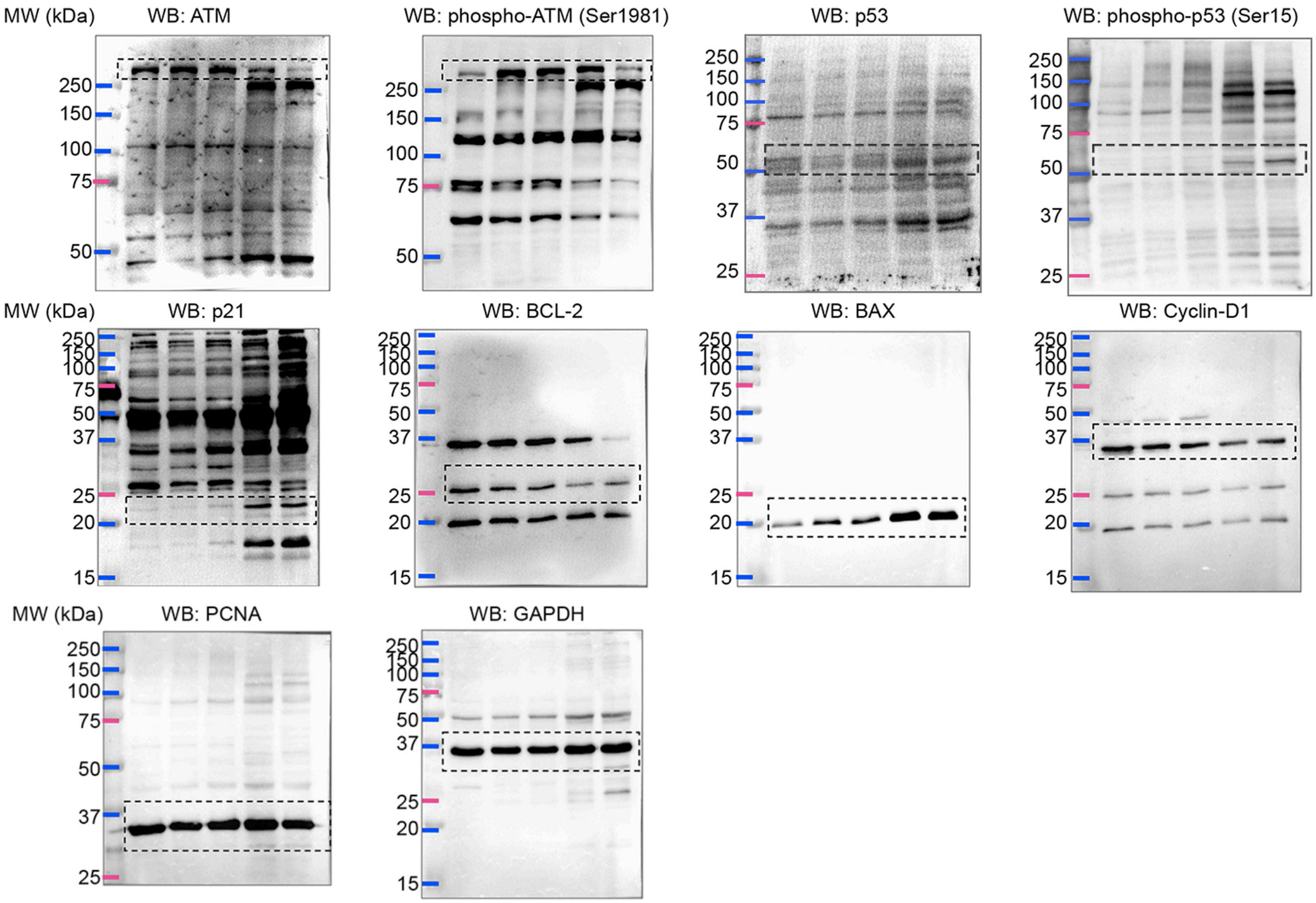

Figure 2k

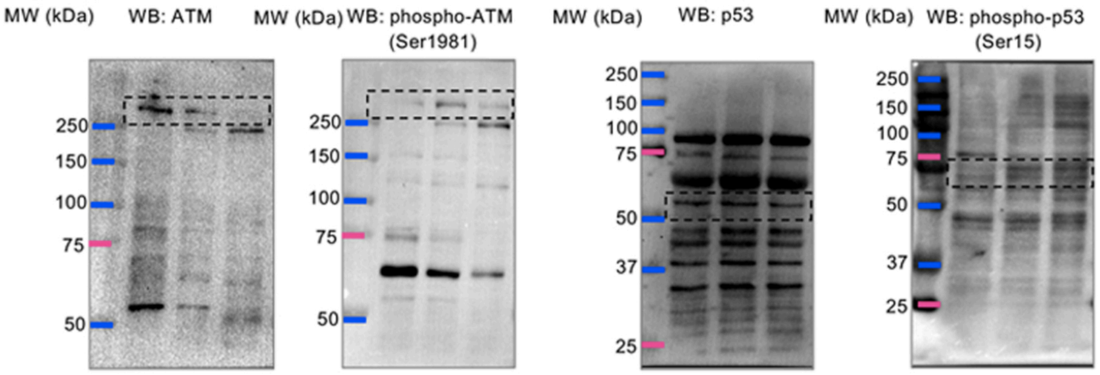

Figure 2l

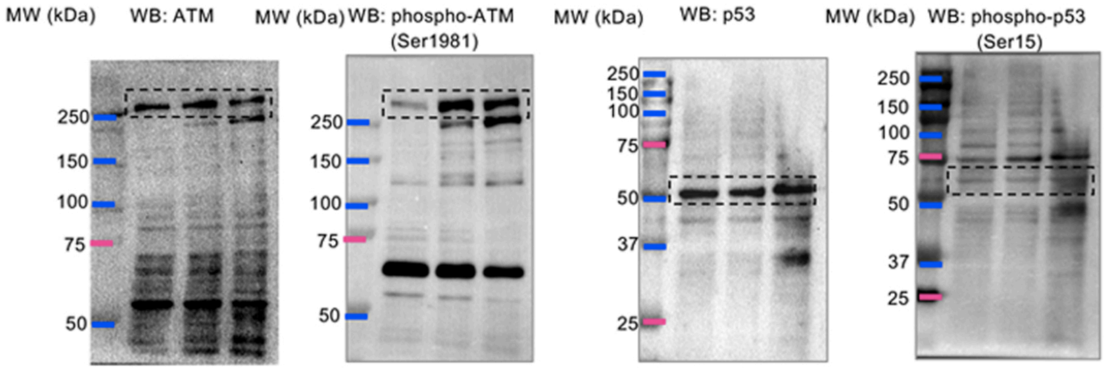

Figure 3k

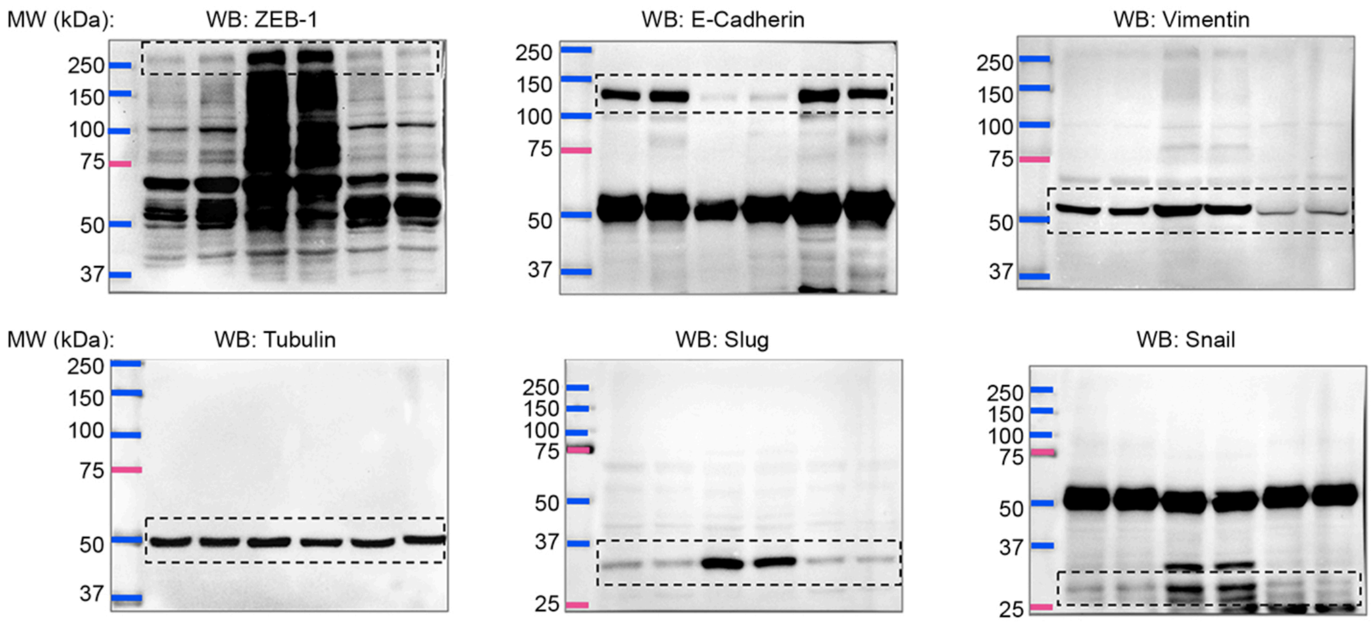

Supplementary Figure 4

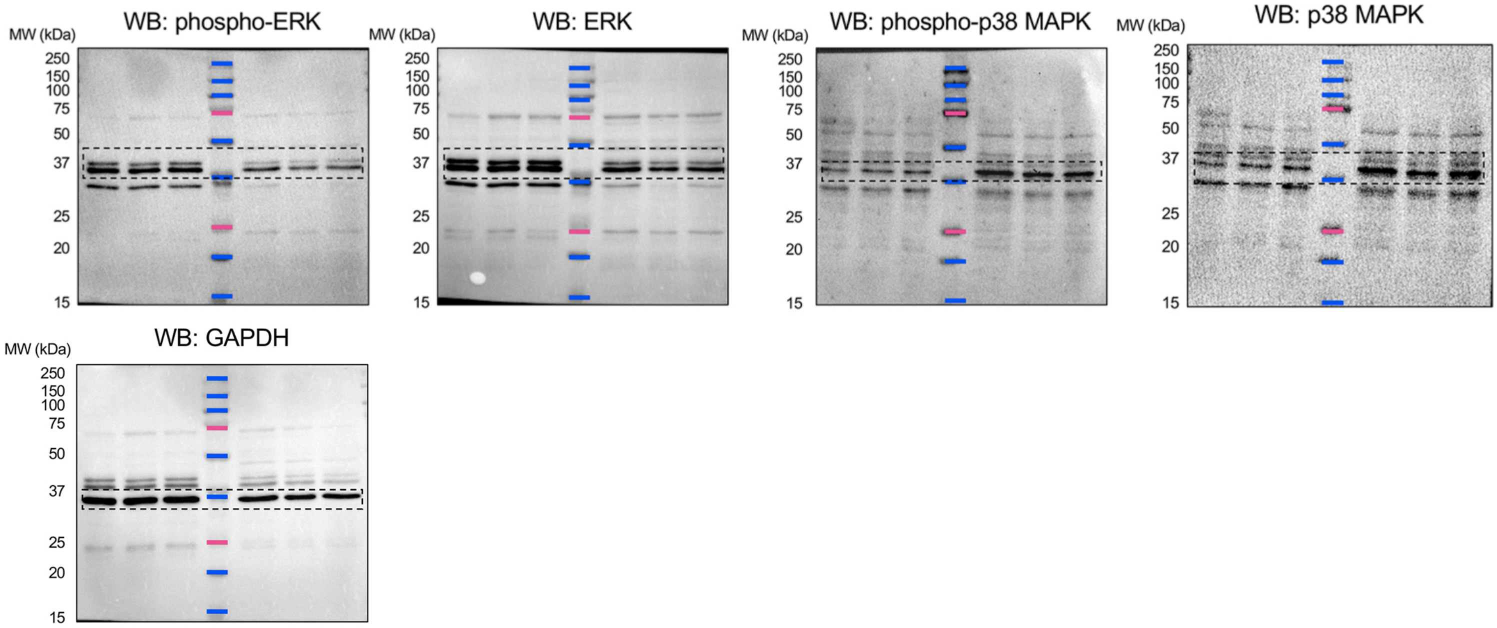

Supplement: Supplementary file 1 — Supplementary Information [file 41467_2023_36755_MOESM1_ESM.pdf]
