# Supplementary material for: Loss of p53 activates thyroid hormone via type 2 deiodinase and enhances DNA damage
Source: Nat Commun. 2023 Mar 4;14:1244. doi: 10.1038/s41467-023-36755-y (PMC9985592; doi:10.1038/s41467-023-36755-y)
Supplement: Supplementary file 2 — Description of Additional Supplementary Files [file 41467_2023_36755_MOESM2_ESM.pdf]

Inventory of Supporting Information

File Name: Supplementary Data 1
Description: Supplementary Data 1 | List of the 111 Differentially Expressed Genes (DEGs) identified from RNA-seq data analysis between p53KO$^{+/-}$;D2KO and p53KO$^{+/-}$;D2WT mice. Genes are listed by Fold Change (FC) order.

File Name: Supplementary Data 2
Description: Supplementary Data 2 | Primers used for Real-Time PCR, ChIP and Dio2-depletion analysis in mouse models.

File Name: Supplementary Data 3
Description: Supplementary Data 3 | List of antibodies indicating the dilution used for Western Blot (WB), Immunofluorescence (IF) and Chromatin Immuno-Precipitation (ChIP) analysis.
