# Supplementary material for: Loss of p53 activates thyroid hormone via type 2 deiodinase and enhances DNA damage
Source: Nat Commun. 2023 Mar 4;14:1244. doi: 10.1038/s41467-023-36755-y (PMC9985592; doi:10.1038/s41467-023-36755-y)
Supplement: Supplementary file 3 — Reporting Summary [file 41467_2023_36755_MOESM3_ESM.pdf]

nature portfolio

Corresponding author(s): Prof. Monica Dentice

Last updated by author(s): Dec 21, 2022

Reporting Summary

Nature Portfolio wishes to improve the reproducibility of the work that we publish. This form provides structure for consistency and transparency in reporting. For further information on Nature Portfolio policies, see our Editorial Policies and the Editorial Policy Checklist.

Statistics

For all statistical analyses, confirm that the following items are present in the figure legend, table legend, main text, or Methods section.

n/a	Confirmed	
☐	☒	The exact sample size (*n*) for each experimental group/condition, given as a discrete number and unit of measurement
☒	☐	A statement on whether measurements were taken from distinct samples or whether the same sample was measured repeatedly
☐	☒	The statistical test(s) used AND whether they are one- or two-sided<br>*Only common tests should be described solely by name; describe more complex techniques in the Methods section.*
☒	☐	A description of all covariates tested
☒	☐	A description of any assumptions or corrections, such as tests of normality and adjustment for multiple comparisons
☐	☒	A full description of the statistical parameters including central tendency (e.g. means) or other basic estimates (e.g. regression coefficient) AND variation (e.g. standard deviation) or associated estimates of uncertainty (e.g. confidence intervals)
☐	☒	For null hypothesis testing, the test statistic (e.g. *F*, *t*, *r*) with confidence intervals, effect sizes, degrees of freedom and *P* value noted<br>*Give P values as exact values whenever suitable.*
☒	☐	For Bayesian analysis, information on the choice of priors and Markov chain Monte Carlo settings
☒	☐	For hierarchical and complex designs, identification of the appropriate level for tests and full reporting of outcomes
☐	☒	Estimates of effect sizes (e.g. Cohen's *d*, Pearson's *r*), indicating how they were calculated

Our web collection on statistics for biologists contains articles on many of the points above.

Software and code

Policy information about availability of computer code

Data collection	Genome Browser, TFBind, GEO Dataset GSE42677, GSEA MSigDB, NCBI SRA PRJNA891519 (https://dataview.ncbi.nlm.nih.gov/object/PRJNA891519?reviewer=6av1frlqu4g53tnrh82rs012j), GEO Dataset GSE42677 (https://www.ncbi.nlm.nih.gov/geo/query/acc.cgi?acc=GSE42677)
Data analysis	ImageJ 1.45s Software, BD FACS Canto Flow Cytometer and Diva Software, ZEISS LSM 900 Airyscan 2, Leica DMi8 Microscope and Leica Application Suite LAS X Imaging Software confocal microscope, Gene Set Enrichment Analysis (GSEA) Software_4.1.0 (gsea-msigdb.org)

For manuscripts utilizing custom algorithms or software that are central to the research but not yet described in published literature, software must be made available to editors and reviewers. We strongly encourage code deposition in a community repository (e.g. GitHub). See the Nature Portfolio guidelines for submitting code & software for further information.

Data

Policy information about availability of data

All manuscripts must include a data availability statement. This statement should provide the following information, where applicable:

- Accession codes, unique identifiers, or web links for publicly available datasets
- A description of any restrictions on data availability
- For clinical datasets or third party data, please ensure that the statement adheres to our policy

Data supporting the findings of this work are available within the paper and its Supplementary Information files. A reporting summary for this Article is available as a

Supplementary Information file. All data from this study have been submitted to the NCBI Sequence Read Archive (SRA). The sequencing data generated for this study have been deposited in NCBI Sequencing Read Archive (SRA) database under the BioProject accession number PRNA891519 (https://www.ncbi.nlm.nih.gov/bioproject/PRJNA891519). The residual dataset used are available in GEO Dataset GSE42677 (https://www.ncbi.nlm.nih.gov/geo/query/acc.cgi?acc=GSE42677). The remaining data are available within the Article, Supplementary Information or Source Data file provided with this paper.

Human research participants

Policy information about studies involving human research participants and Sex and Gender in Research.

Reporting on sex and gender	N/A
Population characteristics	N/A
Recruitment	N/A
Ethics oversight	N/A

Note that full information on the approval of the study protocol must also be provided in the manuscript.

Field-specific reporting

Please select the one below that is the best fit for your research. If you are not sure, read the appropriate sections before making your selection.

☒ Life sciences ☐ Behavioural & social sciences ☐ Ecological, evolutionary & environmental sciences

For a reference copy of the document with all sections, see nature.com/documents/nr-reporting-summary-flat.pdf

Life sciences study design

All studies must disclose on these points even when the disclosure is negative.

Sample size	Sample sizes were determined empirically based on similar studies. For in vitro assays n = 3. For in vivo animal studies n = 12 for survival analyses. The precise number of samples analyzed are indicated in the figure and in the figure legends.
Data exclusions	No data were excluded from the analyses.
Replication	All experiments were performed in triplicate. All attempts at replication were successful.
Randomization	For in vivo experiments, animals were randomly assigned to either control or treatment groups. For in vitro experiments, cells were randomly allocated into different treatment group.
Blinding	Researchers were blinded when collecting data, with image acquisition by one staff member and data analysis by another one. For in vitro experiments, the researchers were blinded to cell genotypes or treatment conditions for imaging and analysis. For RNA-seq experiments, the analyst was blinded to treatment and genotype information.

Reporting for specific materials, systems and methods

We require information from authors about some types of materials, experimental systems and methods used in many studies. Here, indicate whether each material, system or method listed is relevant to your study. If you are not sure if a list item applies to your research, read the appropriate section before selecting a response.

Materials & experimental systems

n/a	Involved in the study	
☐	☒	Antibodies
☐	☒	Eukaryotic cell lines
☒	☐	Palaeontology and archaeology
☐	☒	Animals and other organisms
☒	☐	Clinical data
☒	☐	Dual use research of concern

Methods

n/a	Involved in the study	
☒	☐	ChIP-seq
☐	☒	Flow cytometry
☒	☐	MRI-based neuroimaging

Antibodies

Antibodies used

anti-8-oxo-dG antibody (TREVINGEN, cod. 4354-MC-050)
anti-ATM (abcam, cod. ab199726)
anti-ATM phospho-S1981 (abcam, cod. 81292)
anti-BAX (Cell Signaling, cod. 2772S)
anti-BCL2 (Cell Signaling, cod. 2872S)
anti-CXCR4 (abcam, cod. ab-2074)
anti-Cyclin-D1 (Santa Cruz Biotechnology, cod. sc-246)
anti-Cytokeratin 8 (TROMA 1) Hybridoma bank cod. AB_531826
anti-Cytokeratin 14 (COVANCE, cod. D14IF01918)
anti-E-Cadherin (BD Biosciences, cod. 610181)
anti-ERK1/2 (K-23) (Santa Cruz Biotechnology, cod. sc-94)
anti-FLAG antibody (Sigma-Aldrich, cod. F3165)
anti-GAPDH (Elabscience, cod. E-AB-20059)
anti-mouse IgG-HRP (BioRad, cod. 1706516)
anti-mouse IgG (H+L) Highly Cross-Adsorbed Secondary Antibody, Alexa Fluor 594 (Invitrogen™, cod. A-21203)
anti-mouse IgG (H+L) Cross-Adsorbed Secondary Antibody, Alexa Fluor 488 (Invitrogen™, cod. A-11001)
anti-OGG1/2 (Santa Cruz Biotechnology, cod. sc-376935)
anti-p21 (Santa Cruz Biotechnology, cod. sc-397)
anti-p38 MAPK (Cell Signaling, cod. #9212)
anti-p53 (Cell Signaling, cod. 2524S)
anti-phospho-p44/42 MAPK (ERK1/2) (Thr202/Tyr204) (Cell Signaling, cod. #4370)
anti-phospho-p38 MAPK (Thr180/Tyr182) (Cell Signaling, cod. #9211S)
anti-p53 phospho-Ser15 (Cell Signaling, cod. 9284S)
anti-PCNA (Santa Cruz Biotechnology, cod. sc-7907)
anti-phospho-Histone H2A.X (Ser 139) clone JBW301 (Millipore, cod. 05-636)
anti-rabbit IgG-HRP (BioRad, cod. 1706515)
anti-rabbit IgG (H+L) Highly Cross-Adsorbed Secondary Antibody, Alexa Fluor 594 (Invitrogen™, cod. A-21207)
anti-rabbit IgG (H+L) Cross-Adsorbed Secondary Antibody, Alexa Fluor 488 (Invitrogen™, cod. A-11070)
anti-Rad51 (H-92) (Santa Cruz Biotechnology, cod. sc-8349)
anti-Slug (Cell Signaling, cod. 9585S)
anti-Snail (Cell Signaling, cod. 3895S)
anti-Thyroid hormone receptor antibody (C3)-Chip Grade (abcam, cod. ab-2743)
anti-Thyroid hormone receptor beta antibody Chip Grade (abcam, cod. ab-5622)
anti-Tubulin (Santa Cruz Biotechnology, cod. sc-5546).
anti-Vimentin (abcam, cod. ab-92547)
anti-ZEB1 (abcam, cod. ab-155249)

All the antibodies and the specific dilution used in the study were indicated in Supplementary Data 3.

Validation

All the antibodies are commercially available and validated by the manufacturer.
anti-8-oxo-dG antibody (https://www.bio-techne.com/datasheet-pdf?src=rnd&pdf=4354-mc-050.pdf)
anti-ATM (https://www.abcam.com/atm-antibody-epr17059-ab199726.html)
anti-ATM phospho-S1981 (https://www.abcam.com/atm-phospho-s1981-antibody-ep1890y-ab81292.html)
anti-BAX (https://www.cellsignal.com/products/primary-antibodies/bax-antibody/2772)
anti-BCL2 (https://www.cellsignal.com/products/primary-antibodies/bcl-2-antibody-human-specific/2872)
anti-CXCR4 (https://www.abcam.com/cxcr4-phospho-s339-antibody-ab74012.html)
anti-Cyclin-D1 (https://datasheets.scbt.com/sc-246.pdf)
anti-Cytokeratin 8 (TROMA 1) (https://www.sigmaaldrich.com/IT/it/product/mm/mabt329m)
anti-E-Cadherin (https://www.labome.com/product/BD-Biosciences/610181.html)
anti-ERK1/2 (K-23) (https://datasheets.scbt.com/sc-94.pdf)
anti-FLAG antibody (https://www.sigmaaldrich.com/deepweb/assets/sigmaaldrich/product/documents/274/912/f3165dat-ms.pdf)
anti-GAPDH (https://www.elabscience.com/PDF/Cate98/E-AB-20059-Elabscience.pdf)
anti-OGG1/2 (https://www.scbt.com/it/p/ogg1-2-antibody-g-5)
anti-p21 (https://datasheets.scbt.com/sc-397.pdf)
anti-p38 MAPK (https://www.cellsignal.com/products/primary-antibodies/p38-mapk-antibody/9212)
anti-p53 (https://www.cellsignal.com/products/primary-antibodies/p53-1c12-mouse-mab/2524)
anti-phospho-p44/42 MAPK (ERK1/2) (https://www.cellsignal.com/products/primary-antibodies/phospho-p44-42-mapk-erk1-2-thr202-tyr204-d13-14-4e-xp-rabbit-mab/4370)
anti-phospho-p38 MAPK (Thr180/Tyr182) (https://www.cellsignal.com/products/primary-antibodies/phospho-p38-mapk-thr180-tyr182-antibody/9211)
anti-p53 phospho-Ser15 (https://www.citeab.com/antibodies/125987-9284-phospho-p53-ser15-antibody)
anti-PCNA (https://datasheets.scbt.com/sc-7907.pdf)
anti-phospho-Histone H2A.X (Ser 139) clone JBW301 (https://www.merckmillipore.com/IT/it/product/Anti-phospho-Histone-H2A.X-Ser139-Antibody-clone-JBW301,MM_NF-05-636)
anti-Rad51 (H-92) (https://datasheets.scbt.com/sc-8349.pdf)
anti-Slug (https://www.cellsignal.com/products/primary-antibodies/slug-c19g7-rabbit-mab/9585)
anti-Snail (https://www.cellsignal.com/datasheet.jsp?productId=3895&images=1)
anti-Thyroid hormone receptor antibody (C3)-Chip Grade (https://www.abcam.com/thyroid-hormone-receptor-antibody-c3-chip-grade-ab2743.html)
anti-Thyroid hormone receptor beta antibody Chip Grade (https://www.abcam.com/thyroid-hormone-receptor-beta-antibody-ab5622.html)
anti-Tubulin (https://datasheets.scbt.com/sc-5546.pdf)

anti-Vimentin (https://www.abcam.com/vimentin-antibody-epr3776-cytoskeleton-marker-ab92547.html)
anti-ZEB1 (https://www.abcam.com/zeb1-antibody-ab155249.html)

Eukaryotic cell lines

Policy information about cell lines and Sex and Gender in Research

Cell line source(s)	SCC011 (RRID:CVCL_5986); SCC13 (RRID:CVCL_4029); HaCaT (RRID:CVCL_0038)
Authentication	Cell line were authenticated by specific cell morphology
Mycoplasma contamination	All cell lines were mycoplasma free
Commonly misidentified lines (See ICLAC register)	No misidentified lines were used in the study.

Animals and other research organisms

Policy information about studies involving animals; ARRIVE guidelines recommended for reporting animal research, and Sex and Gender in Research

Laboratory animals	K14-CreERT;p53WT;D2WT; K14-CreERT;p53KO+/-;D2fl/fl and D2-3x_Flag mice were C57/B16 strain. 2-months-old mice were used. All the experiments were performed in accordance with institutional guidelines. All mice were maintained under standard conditions, at ambient temperature, 60% humidity, 12-hour light/dark cycles and received a standard diet and water ad libitum. Mice were euthanized by CO2 exposure followed by cervical dislocation when tumor diameter reached the maximal tumor size allowed by the IACUC committee.
Wild animals	No wild animals were used.
Reporting on sex	Only male mice were used in this study.
Field-collected samples	No field-collected samples were used.
Ethics oversight	All animal experiments were conducted in accordance with the guidelines of Ministero della Salute and were approved by the Institutional Animal Care and Use Committee (IACUC, n. 167/2015-PR and 354/2019-PR).

Note that full information on the approval of the study protocol must also be provided in the manuscript.

Flow Cytometry

Plots

Confirm that:

☒ The axis labels state the marker and fluorochrome used (e.g. CD4-FITC).

☒ The axis scales are clearly visible. Include numbers along axes only for bottom left plot of group (a 'group' is an analysis of identical markers).

☒ All plots are contour plots with outliers or pseudocolor plots.

☒ A numerical value for number of cells or percentage (with statistics) is provided.

Methodology

Sample preparation	For apoptosis detection, cells were resuspended in PBS, fixed with ice-cold ethanol, and treated with RNase and Propidium Iodine (PI) and analyzed on a BD FACS Canto Flow Cytometer (BD Bioscience).
Instrument	BD FACS Canto Flow Cytometer (BD Bioscience).
Software	BD FACS Diva Software.
Cell population abundance	Sort-purification was carried out using acustom configuration FACS Canto2 cell sorter.
Gating strategy	Living cells were selected by forward scatter, side scatter and doublet discrimination by Hoechst dye exclusion.

☒ Tick this box to confirm that a figure exemplifying the gating strategy is provided in the Supplementary Information.
